# Error-Robust Modes of the Retinal Population Code

**DOI:** 10.1371/journal.pcbi.1005148

**Published:** 2016-11-17

**Authors:** Jason S. Prentice, Olivier Marre, Mark L. Ioffe, Adrianna R. Loback, Gašper Tkačik, Michael J. Berry

**Affiliations:** 1 Princeton Neuroscience Institute, Princeton University, Princeton, NJ, United States of America; 2 Institut de la Vision, UMRS 968 UPMC, INSERM, Paris, France; 3 Physics Dept., Princeton University, Princeton, NJ, United States of America; 4 Institute of Science and Technology Austria, Austria; 5 Molecular Biology Dept., Princeton University, Princeton, NJ, United States of America; University of Connecticut, UNITED STATES

## Abstract

Across the nervous system, certain population spiking patterns are observed far more frequently than others. A hypothesis about this structure is that these collective activity patterns function as population codewords–collective modes–carrying information distinct from that of any single cell. We investigate this phenomenon in recordings of ∼150 retinal ganglion cells, the retina’s output. We develop a novel statistical model that decomposes the population response into modes; it predicts the distribution of spiking activity in the ganglion cell population with high accuracy. We found that the modes represent localized features of the visual stimulus that are distinct from the features represented by single neurons. Modes form clusters of activity states that are readily discriminated from one another. When we repeated the same visual stimulus, we found that the same mode was robustly elicited. These results suggest that retinal ganglion cells’ collective signaling is endowed with a form of error-correcting code–a principle that may hold in brain areas beyond retina.

## Author Summary

Neurons in most parts of the nervous system represent and process information in a collective fashion, yet the nature of this collective code is poorly understood. An important constraint placed on any such collective processing comes from the fact that individual neurons’ signaling is prone to corruption by noise. The information theory and engineering literatures have studied error-correcting codes that allow individual noise-prone coding units to “check” each other, forming an overall representation that is robust to errors. In this paper, we have analyzed the population code of one of the best-studied neural systems, the retina, and found that it is structured in a manner analogous to error-correcting schemes. Indeed, we found that the complex activity patterns over ~150 retinal ganglion cells, the output neurons of the retina, could be mapped onto collective code words, and that these code words represented precise visual information while suppressing noise. In order to analyze this coding scheme, we introduced a novel quantitative model of the retinal output that predicted neural activity patterns more accurately than existing state-of-the-art approaches.

## Introduction

Understanding the manner in which population of neurons encode information is fundamental to systems neuroscience. Recent years have seen rapid progress in experimental techniques for recording simultaneously from hundreds of neurons of more [1–5], which provide us with excellent access to the activity states of relevant neural populations. However, what continues to make this problem challenging and mathematically complex is the fact that collective neural activity patterns have enormous dimensionality–for instance, if we only need to keep track of spiking or silence for each neuron, a population of *N* neurons still has 2^*N*^ possible activity states [6, 7]. While debate rages about the amplitude and significance of noise correlations [7–10], it is well established that nearby neurons have overlap in their receptive fields or tuning curves which introduces signal correlation and redundancy. Notwithstanding the popularity of ideas about efficient coding and redundancy reduction [11, 12], direct measurement reveals extensive information redundancy among neurons in many brain regions [13–17].

One popular framework has been *ring models* or *probabilistic population codes*, in which a population of neurons with a spectrum of different tuning curves encode for scalar stimulus variables, such as neurons in V1 encoding the orientation of a bar of light [18–21]. Study of these models has revealed many important insights into population codes, such as the dramatic effect that weak pairwise correlations can have on the code of a large neural population as well as the sensitivity of these effects to the specific pattern of correlation among neurons [7, 18, 22–24]. However, the generalization of this framework to the high-dimensional stimuli that we often encounter in real life is not so obvious.

An alternative approach is to formulate approximate models of the probability distribution over all possible neural activity patterns [25–27] and examine the structure of this entire probability landscape. An appealing hypothesis about the function of large populations of sensory neurons is that their combinatorially huge space of possible codewords could enable a form of error correction. Specifically, the high capacity *N*-dimensional coding space could be partitioned into subsets, and within each subset distinct words would be considered noise-corrupted versions of one another. The actual information conveyed by the channel is then the identity of the relevant subset, as opposed to the precise word within that subset. This qualitative picture is the intuitive basis of error-correcting codes [28]; it is natural to then ask whether the redundancy observed in local neural circuits is a signature of such codes being operative in the neural population.

In the retina, each image point is encoded by many ganglion cells with overlapping receptive fields [29–31]. The population code of the retinal ganglion cells is understood as having parallel channels of visual information: each channel is formed by ganglion cells of a single functional/morphological type, whose receptive fields tile visual space with minimal overlap, and each such mosaic conveys qualitatively different visual information to the brain using receptive fields with different spatiotemporal selectivity [32]. The simplest idea is that different visual channels project to different targets in the central brain, thus subserving different visual behaviors. However, there are many ganglion cell types, at least 32 [31], and only two major targets in the central brain: the LGN and the superior colliculus. Furthermore, most ganglion cell axons branch and project to multiple targets [33]. In fact, almost all of the ganglion cells send collaterals to the superior colliculus / optic tectum in many species [34–37].

One might argue that different ganglion cell types segregate to different lamina in thalamus [38] and colliculus [39], thereby keeping the visual channels distinct. However, these channels recombine by synapsing into the same target neurons as early as layer 2/3 of the primary visual cortex [40] and the intermediate layers of the colliculus [41]. Therefore, visual centers just beyond the retina are able to combine information across the different parallel channels. Important for the scope of population codes, correlation is significant between nearest neighbor ganglion cells in each mosaic [42–44] as well as among ganglion cells of different functional type [13, 30, 45]. And combinations of firing among multiple ganglion cells have been shown to encode different visual features than those encoded by the constituent cells [46–48]. As a result, populations of hundreds of neurons from a combinatorial neural code as early as the level of the retinal ganglion cells.

Since downstream areas infer visual information solely from the ganglion cell spike trains, this choice of readout must be derived from the statistical structure of the retinal population output. The question of optimal statistical structure over noisy, spiking sensory neurons has been investigated theoretically [49]. In conditions of high noise, an error-correcting organization featuring discrete clusters of population words optimizes information transfer. Is this the case in real neural populations? In the present paper, we address this question empirically by fitting a statistical model to the retinal ganglion cell population representing a small visual patch, and show that a discrete population structure emerges from the model and naturally captures qualitatively important features of the visual stimulus.

We obtained multi-electrode array data from ∼150 salamander retinal ganglion cells simultaneously responding to various visual stimuli, including natural movies. Our electrode array covered a small, dense patch of retina, so that the cells in our recording had largely overlapping receptive fields conveying information about a restricted region of visual space [1]. We then investigated a model in which the complex, fluctuating patterns of spikes across the population could be grouped into a modest number of “collective modes.” The possibility of this dimensionality reduction is a consequence of redundancy in the retinal code. Our model is highly tractable to fit, and allows for exact sampling from the probability distribution over retinal population response patterns. We found that this model provided an excellent description of the frequency of occurrence of activity patterns in the neural population, and we further investigated its implications for information encoding. After fitting the model, we inferred the temporal sequence of mode occurrence underlying the data. Population modes carried a detailed representation of the visual stimulus. Moreover, our model grouped spiking patterns together into discriminable modes in a manner reminiscent of error-correcting codes, and the modes were indeed far more reliable over repeated stimulus presentations than were individual spiking patterns. Thus, the organization of the activity states into collective modes may constitute an important aspect of the population neural code of retinal ganglion cells. We also note that nothing in our analysis methods was specific to the retina, and so this approach can be readily applied to population codes in other brain regions as well.

## Results

### Decomposing the activity states of the retinal ganglion cell population into collective modes

**Fig 1:**
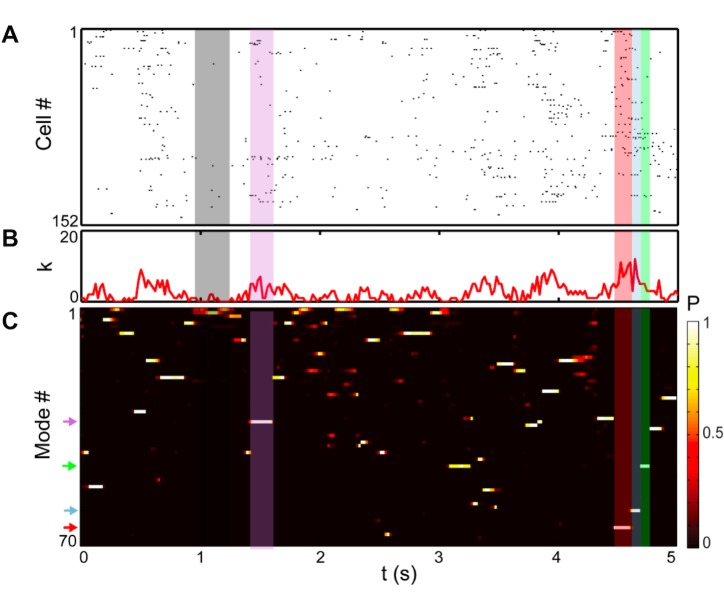
Decomposing retinal population responses into modes. (A) Example spike raster from 5 sec of training data (*N* = 152 cells). Each column represents the population spiking pattern as a binary word, {*σ*_*i*_(*t*)}, defined over a 20 ms time bin. Colored bands indicate periods of time during which a particular mode was active. (B) Population spike count. Note that activity is characterized by bouts of highly correlated population spiking punctuated by periods of relative quiescence. (C) Time-varying probability, *P*, of each mode, given all the observed data. *P* is quantified by fitting the model described in Fig 2 and in the main text. Modes are sorted top-to-bottom by decreasing probability. Note that periods of high population spike count ($k=\sum_{i}\sigma_{i}$) generally corresponded to well-defined modes (*P* of one mode near 1, with sharp temporal transitions between modes), while periods when *P* was smeared across several modes coincided with the population being more quiescent (grey band in B).

We recorded spike trains from 152 retinal ganglion cells in response to a non-repeated natural movie stimulus (see Methods). In our recordings, the retinal population response was characterized by rapidly fluctuating groups of ∼10 neurons spiking simultaneously, interspersed with periods of relative quiescence (Fig 1A and 1B). If such patterns involve specific subsets of neurons firing together more regularly than expected by chance, they may represent distinct population code words that capture collective information not represented by single-cell responses alone. In this paper, we refer to these patterns as “collective modes” of the retinal ganglion cell population, and we investigate a model that explicitly captures their structure.

A natural way to incorporate such structure into a statistical model is by assuming the existence of a discrete set of hidden states representing the set of collective modes in the population, which we denote by the symbol *α*. Modes were therefore described by a latent variable of our model that changed across time, *α*(*t*). The observed pattern of population spiking at a given time then depends on the instantaneous mode present at that time. Since the observed spiking pattern depends upon unobserved stimulus- and noise-driven fluctuations, the spiking pattern {*σ*_*i*_(*t*)} will be generated *probabilistically* by the mode *α*(*t*). To model this relationship quantitatively, we introduced for each mode a probability distribution over spiking patterns, *Q*_*α*_({*σ*_*i*_}). This is the conditional distribution of the spike pattern given the mode, and is termed the mode’s *emission distribution*.

The mean of this distribution assigns to each cell, *i*, a mode-dependent firing probability *m*_*iα*_ = *E*[*σ*_*i*_|*α*], where *σ*_*i*_ = 1 if cell *i* spiked in a time bin, and *σ*_*i*_ = 0 otherwise (see Methods). By associating to each mode a unique pattern of firing probability, with different subsets of cells likely to be active in different modes, many spiking patterns can be combined in a flexible way. Therefore, a latent variable model can, in principle, capture arbitrary patterns of high-order correlation among cells, even without incorporating complex correlations into the emission distributions *Q*_*α*_({*σ*_*i*_}). However, we found that model performance was improved by the addition of weak mode-dependent correlations (see below, and Methods). After learning the model parameters (see Methods), we could then invert the model to infer the probability of each mode as a function of time (Fig 1C). Our techniques therefore allowed us to transform the observed population spike raster (Fig 1A) into a simpler representation, the temporal sequence of modes (Fig 1C).

**Fig 2:**
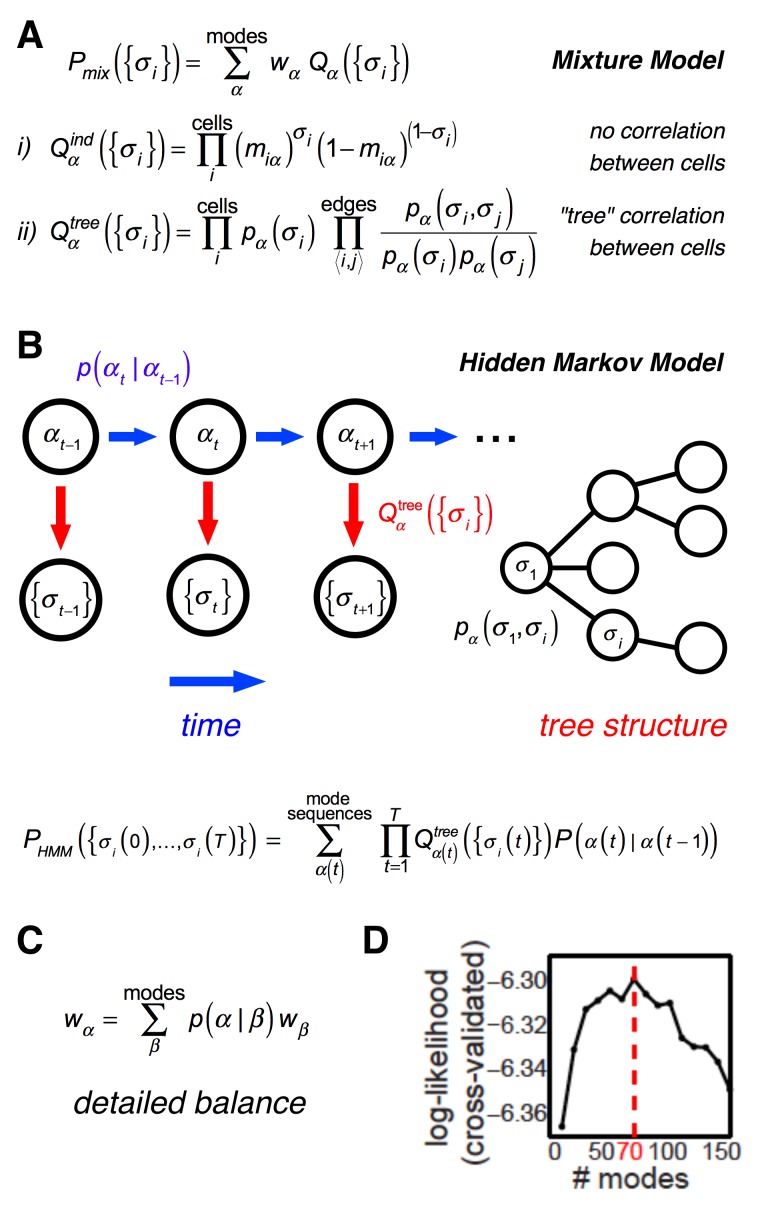
Description of the structure of the hidden Markov model (HMM). (A) In the limit of no temporal correlations, the probability over all binary spike words is given by a mixture over different emission distributions. (i) In the case of no correlation within an emission distribution, *Q*^*ind*^ has a simple product form; (ii) our full model includes pairwise correlations within an emission distribution, *Q*^*tree*^, having a tree graphical structure. (B) *Left*: The full model also includes temporal correlation through a transition probability matrix, *P*(*α*(*t*)|*α*(*t*−1)), which describes the probability of finding mode *α* at time step *t* given the mode present in the previous time step, *t-1*. The hidden state (mode identity), *α*, evolves probabilistically over time according to the transition matrix, and the observed pattern of spiking at time *t*, {*σ*_*i*_(*t*)}, has a mode-dependent emission probability, $Q_{\alpha }^{\text{tree}}$. *Right*: Schematic representation of the tree graphical structure of each emission distribution. (C) In order to find the stationary distribution of the HMM, we solved for the set of mode weights, {*w*_*α*_}, using the detailed balance equation. (D) The total number of modes, *M*, was determined by finding the maximum 2-fold cross-validated likelihood of the model. Note that the optimum was relatively shallow, so that the model is not sensitive to the precise number of modes, as long as it lies within a certain range (for this dataset, roughly 30–100 modes).

Formally, we modeled population spiking activity with a hidden Markov model [50] (Fig 2). In order to better understand the full structure of this model, it is instructive to introduce features of the model sequentially. In the limit in which there are no temporal dependencies, this form reduces to a mixture model, in which the static probability distribution is described as a weighted sum over a set of emission distributions (Fig 2A). If there are no mode-dependent correlations among cells, then emission distributions are simply a product over each mode-dependent response probability (Fig 2A, *i*). However, we can capture some of the effects of neural correlation given a particular mode by adding pairwise correlations having a tree structure to the emission distribution (Fig 2A, *ii*). These pairwise correlations are characterized by a joint response probability, *p*_*α*_(*σ*_*i*_,*σ*_*j*_). We can represent this emission distribution with a graph having nodes for each cell and edges for each joint probability (Fig 2B). This graph has the structure of a tree, meaning that there are no loops formed by the edges of the graph.

Our full model included temporal correlations within ganglion cell spike trains through a non-uniform probability to transition from one mode to another mode in the subsequent time bin (Fig 2B). This probability of a transition between modes in adjacent time bins, *P*(*α*(*t*)|*α*(*t*−1)), is termed the *transition matrix*. The model was completed by specifying the emission distributions, *Q*_*α*_({*σ*_*i*_}). We found that the simplest choice of independent emission probabilities predicted too-small correlations between cells, necessitating the inclusion of correlations into the emission probabilities. In order to incorporate weak correlations while still maintaining tractability of the model, we parameterized the emission probability distributions by constraining joint distributions on pairs of neurons chosen to form a tree (Fig 2B). With this choice, correlation coefficients between neuron pairs fall off exponentially with the number of tree links separating the two neurons (see Supporting Information File). Most neuron pairs were thus only weakly correlated within a given mode, and the overall correlation structure was captured by the fluctuating hidden mode. Intuitively, one can think of the visual stimulus as determining which mode is activated, in which case the tree structure represents noise correlation while the presence of multiple emission distributions induces signal correlation. For some purposes, we would like our model to estimate a static (time-independent) probability distribution. To this end, we calculated the weights, {*w*_*α*_}, that solved the detailed balance equation (Fig 2C) and then used the mixture model formed with the fitted emission distributions (Fig 2A).

**Fig 3:**
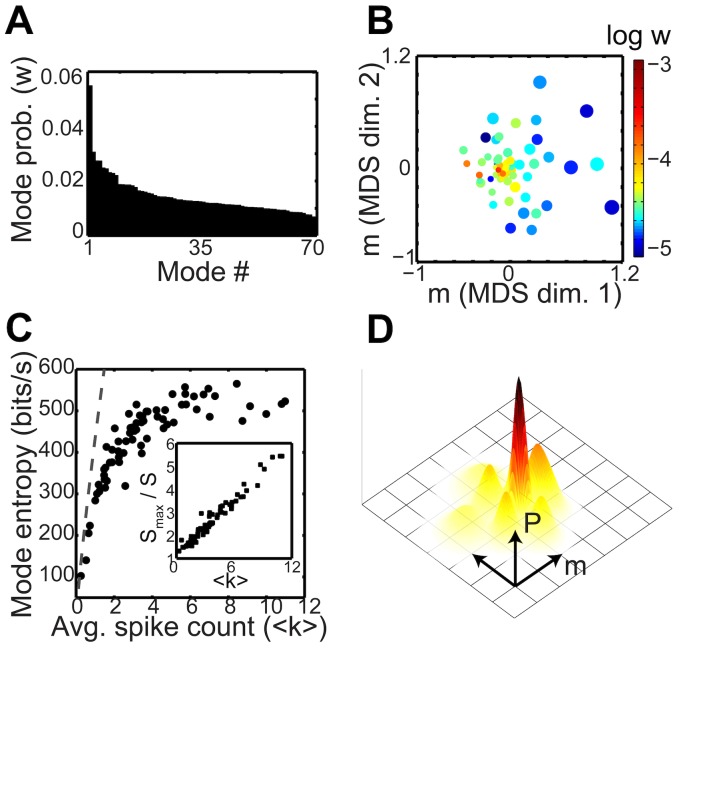
Visualization of model structure. (A) Overall mode weight, *w*_*α*_, versus mode index. (B) The mean spiking probability of each mode, *m*_*iα*_, projected into two dimensions by multidimensional scaling (MDS). The color of each point indicates that mode’s log probability (as in A) while the size scales with the mode’s average population spike count, ⟨*k*⟩. Modes with higher ⟨*k*⟩ pack response space less densely. (C) Entropy rate of mode emission distribution, *S*_*α*_ (see Methods), plotted against average population spike count of the mode. Dashed gray line: *Smax*, the maximum entropy for fixed *k*; i.e. the entropy of the uniform distribution over all *k*-spike words (see Methods). *Inset*: the ratio of maximum entropy to mode entropy. (D) Schematic representation of the structure of the response probability distribution. Each lump represents one mode, with the overall amplitude decreasing and width increasing with average response magnitude, *m*. The tall, narrow, central peak corresponds to the silent and near-silent words near *m* = 0.

**Fig 4:**
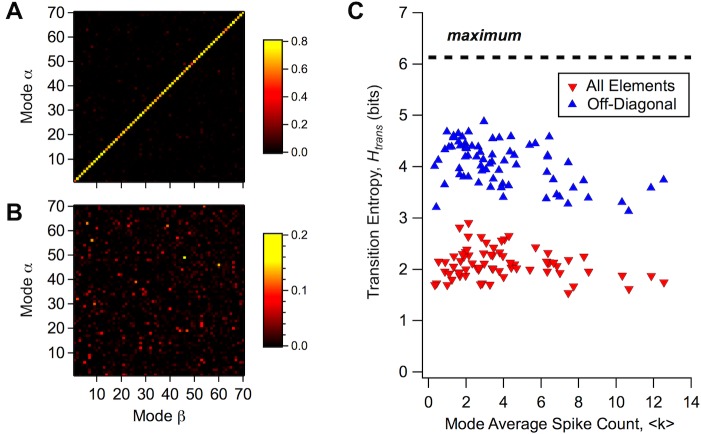
The matrix of transition probabilities between modes. (A) Values of the transition matrix, *P*(*α* | *β*), which describes the probability of finding mode α at time step *t* given the presence of mode *β* at time step *t-1* (color scale). Notice that with *M* = 70 modes, this is a 70x70 matrix. (B) Same transition matrix as in A but with only off-diagonal elements displayed. (C) Transition entropy, *H*_*trans*_(*β*), of each mode plotted against the mode’s average firing rate, ⟨*k*⟩, for the full transition matrix (red) and for the renormalized matrix containing only the off-diagonal elements (blue); maximum possible transition entropy (dashed line).

**Fig 5:**
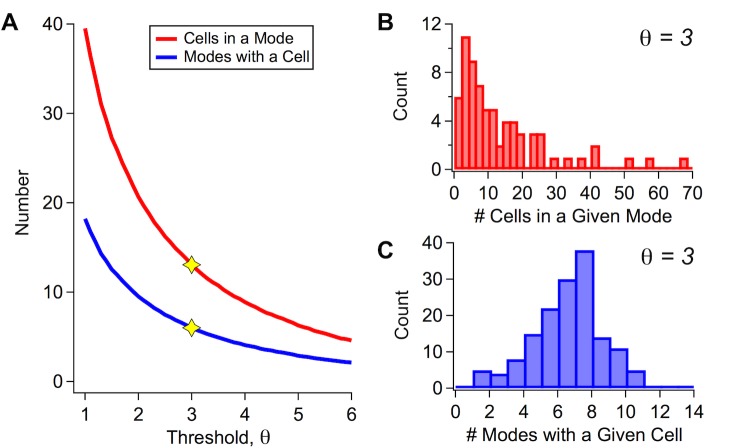
Organization of ganglion cells within collective modes. (A) Average number of cells within a given mode (red) and average number of modes containing a given cell (blue) plotted as a function of the firing threshold, *θ*. (B) Histogram of the number of cells within a given mode for *θ* = 3. (C) Histogram of the number of modes containing a given cell for *θ* = 3.

**Fig 6:**
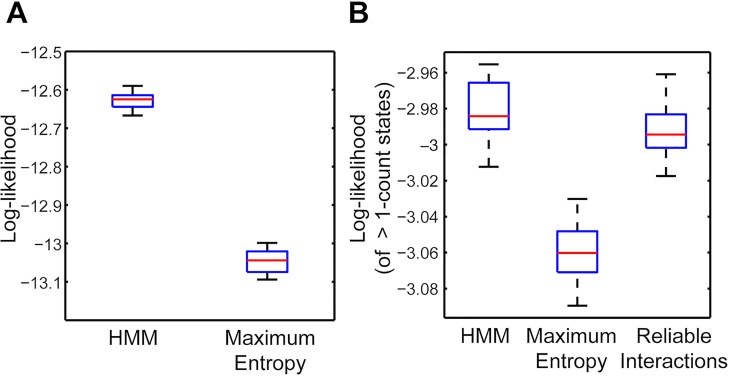
Performance of hidden Markov model (HMM) compared to other models. Performance was assessed by the log-likelihood evaluated on a 50% held-out test set. We generated 10 random 50% train-test splits; the box plots indicate the distribution of test log-likelihood over these splits. Red line = median; blue box = 25% (lower) and 75% (upper) percentiles; black lines = minimum (lower) and maximum (upper). (A) The HMM outperformed the K-pairwise maximum entropy model. (B) Comparison of the HMM, the same maximum entropy fits as *A*, and the reliable interactions model constrained on words occurring at least twice in the train set. Note that the reliable interactions model is a pseudo-likelihood model and therefore does not allow calculation of a meaningful log-likelihood: we therefore followed the convention established in [52] and calculated the log-likelihood summed only over words occurring at least twice in the data. In panel (B), we compare the same truncated log-likelihood across all three models.

**Fig 7:**
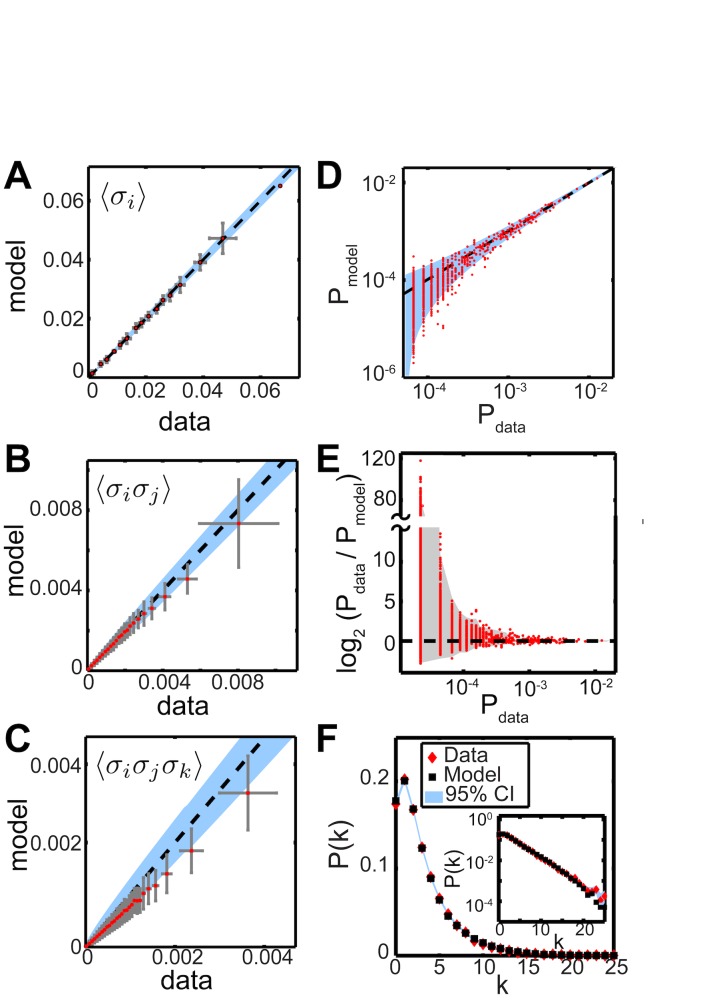
Performance of model in reproducing spiking statistics. (A – C) Low-order moments predicted by the model (y-axis) vs. measured in the data (x-axis). Each data point is an average over an adaptive bin chosen to contain at least 100 cell pairs (B), or triplets (C). Error bars represent ±1 standard deviation. Shaded blue band indicates the 95% confidence interval. (D) Model prediction of individual word probabilities. Words that occurred fewer than 4 times in the data are omitted, as is the all-silent state (whose predictive accuracy is visible in (F)). The blue band indicates the 95% confidence interval, dashed line represents equality. (E) Comparison of model-predicted with empirical word frequencies, extended to include all observed words except the silent state. The gray band represents the range of values for synthetic data sampled from the fit model, with the number of samples matched to the test set (parametric bootstrap). This exhibits almost as much scatter as the true data, even though the model is exactly correct in this synthetic case. (F) Model prediction of population spike-count distribution *P*(*k*), where $k=\sum_{i}\sigma_{i}$. *Inset*: same data as main panel, but with the y-axis log-scaled. Note the close match in the tail of the distribution, which corresponds to very rare events. All goodness-of-fit statistics displayed in this figure were calculated on a 50% held-out test set.

**Fig 8:**
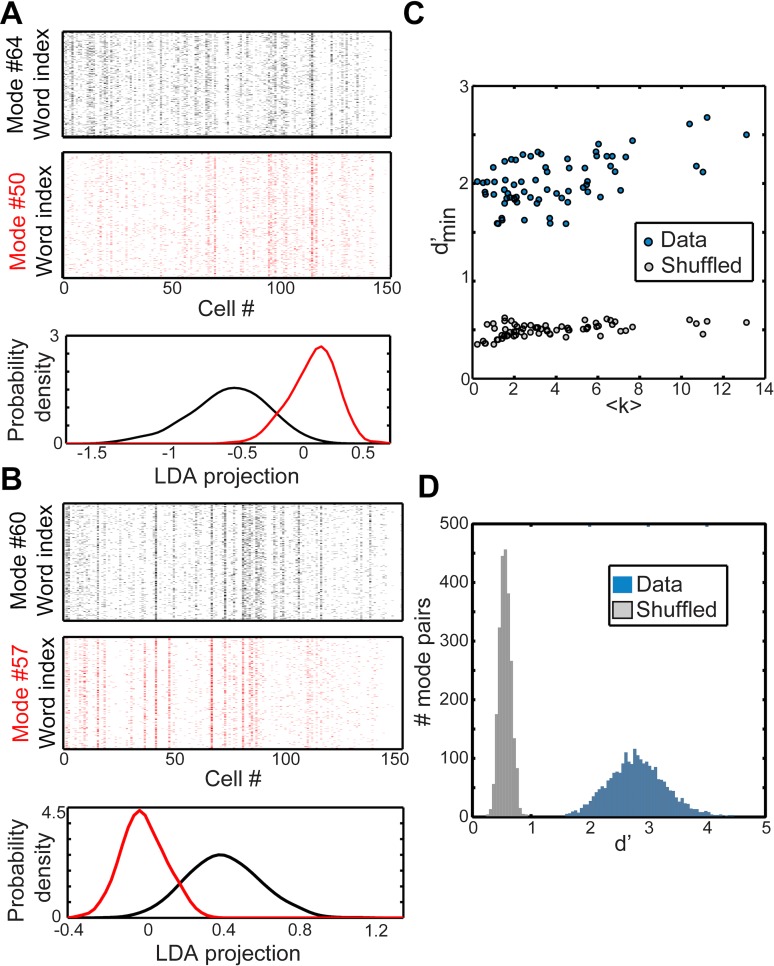
Discriminability of modes. (A,B) Discriminability of some example modes. For each mode, we collected all response words assigned to that mode (A-B, *top*). Words that occurred within one time bin of a transition between two modes were omitted. For each pair of modes, we then carried out Fisher’s linear discriminant analysis (LDA) to find the one-dimensional projection of the two word populations that maximized their discriminability (quantified by *d*′). A and B each summarize this analysis for an arbitrarily chosen example mode (black) and its nearest-neighbor (the mode with smallest *d*′), in red. *Top*: Rasters of unique words assigned to each of the two modes (rows indexing words, columns cells). *Bottom*: Distribution of LDA projections of the words shown above. (C) Blue points: *d*′ of nearest-neighbor modes. Discriminability is high and increases with *⟨k⟩*, consistent with the suggestion of panels A and B. Gray points: results of the same analysis carried out on shuffled data in which words were randomly assigned to modes, keeping the same number of words in each mode. Shuffled modes are highly overlapping, even though the LDA projection maximizes *d*′. (D) Blue: distribution of *d*′ for all mode pairs, not just nearest neighbor. Gray: result on shuffled data, as in (C).

A latent variable model provides great flexibility in capturing arbitrary dependency structures, but the costs of this flexibility are (i) the possibility of overfitting to training data, and (ii) of needing an intractably large number of hidden states to accurately model the data. As an extreme example, experimental data from *N* neurons could be perfectly reproduced by a model with 2^*N*^ hidden states, one for each possible word. We therefore controlled overfitting by selecting the number of modes by a cross-validation procedure (Fig 2D). For the natural movie recording presented in Figs 1–8, 70 modes optimized the cross-validated likelihood. We note that there were fewer modes than cells (*N* = 152 cells in this data set), suggesting that the complexity of the model would remain relatively low even for large populations.

### Qualitative structure of the model

We next analyzed the fitted parameters of the model in order to understand the structure of the response probability distribution and its decomposition into modes. The structure and distribution of the modes may be described at a high level by three quantities per mode: the overall probability weight, the centroid location, and the size. There was one mode with significantly higher probability than the others; this corresponded to the mode with the highest emission probability for the all-silent state. The remaining modes had roughly similar probability to one another (Fig 3A).

In addition to its overall probability *w*_*α*_, each mode has a location in response space given by its mean spike probability vector, *m*_*iα*_ = *E*[*σ*_*i*_|*α*]. To visualize the distribution of these *N*-dimensional centroid vectors, we projected them into two dimensions by applying multidimensional scaling (MDS). This projection preserves the location of the zero point, and approximately preserves Euclidean distance between points. Therefore, radial distance from zero in the MDS plane closely corresponds to the overall activity level of the mode. Modes were found dispersed throughout the MDS plane, roughly tiling most of this space. Notably, the high-activity modes appeared to pack less densely than the low-activity modes (Fig 3B).

Finally, we sought to examine the width of each mode, which we quantified by the entropy of the emission distribution, *S*_*α*_ (see Methods). This measure increased with the modes’ average population spike count ⟨*k*⟩, suggesting that the modes occupied an expanding region of response space at higher activity levels (Fig 3C). Notice that this property is complementary to the observation that high-activity modes pack the MDS space less densely. The mode identity code therefore became coarser with increasing spike count ⟨*k*⟩. However, the tremendous increase in the number of possible activity patterns at higher ⟨*k*⟩ potentially compensate for this coarse-binning (Fig 3C, dashed line). Indeed, the total amount of response capacity increased faster than the entropy per mode (Fig 3C, *inset*). Therefore, provided sufficiently non-overlapping mode distributions, this high capacity of activity patterns at higher ⟨*k*⟩ could be partitioned into a larger number of discriminable modes than at lower ⟨*k*⟩. The increasing size of these regions could then support noise suppression, as in error-correcting codes.

A schematic depiction of the structure implied by examining the parameters of our model is shown in Fig 3D. The probability distribution resembles a “mountain” with an overall peak given by the all-silent state and an overall slope that corresponds to a decrease in the probability as the spike count increases due to the sparseness of neural activity. Extending out from the central peak are localized lumps of probability—peaks and ridges—that correspond to each mode. Each local lump decreases in amplitude and increases in area as the overall activity level increases. Because the volume of response space increases with activity level (represented by rings around the central peak with increasing circumference), the number of modes increases with activity level.

Another important element of our HMM is the matrix of transition probabilities, *P*(*α* | *β*), which describes the probability of finding mode *α* at time step *t* given the presence of mode *β* at time step *t-1*. This matrix was dominated by the diagonal elements (Fig 4A), which introduced a persistence of each mode across several time bins (20 ms). Because the temporal kernel of ganglion cell receptive fields typically has a width of ~100 ms [30], it is expected that the activity state of the ganglion cell population exhibits persistence on this time scale. The median probability of remaining in the same mode on a subsequent time step was 0.70. The average dwell time spent in the same mode ranged from 20 ms to 77 ms. We also found weaker, distributed off-diagonal transition elements (Fig 4B). These off-diagonal elements reflect temporal correlations in the stimulus and are expected to depend strongly on the choice of stimulus ensemble.

In order to assess the structure and significance of the off-diagonal transition elements, we calculated the transition entropy, *H*_*trans*_(*β*), which measures how many modes can be accessed when starting from mode *β* (defined in Methods). This transition entropy had a value of ~2 bits, which implies that the number of accessible states was $2^{H_{trans}}$ ≈ 2^2^ = 4 (Fig 4C). If we removed the diagonal transition element and renormalized the probabilities, we found a much higher number of accessible states, ~2^4^ = 16 (Fig 4C), consistent with the fact that the diagonal transition element always dominated. The transition entropy was roughly constant across different modes, and there were no modes with exceptionally low values. These results are consistent with a picture in which each mode eventually transitions to a broad set of other possible modes, and hence there were no strong ‘sequences’ of modes preferentially observed in our data.

Finally, we can gain more intuition about the structure of our model by investigating how individual ganglion cells are organized into collective modes. One important measure of this organization is the number of individual cells that participate in each collective mode. Another complementary measure is the number of modes containing a given cell. Because each cell has a mode-dependent firing probability, *m*_*iα*_, that varies continuously from zero to one, both of these measures are subject to an arbitrary choice of how large *m*_*iα*_ must be in order for a cell to “participate” in that mode. Thus, we calculated these measures as a function of the threshold criterion, $\theta =m_{i\alpha }/{\bar{m}}_{i}$, where ${\bar{m}}_{i}$ is the average firing probability for cell *i* across all the modes (Fig 5A). At *θ* = 0, these values matched the total number of cells and modes, respectively, and they decayed monotonically to zero for large *θ*. At a threshold of *θ* = 3, the number of cells participating in a given mode showed a highly skewed distribution with an average of 13. Another way of viewing these statistics is to note that the majority of the modes had 10 or fewer cells participating, and less than 30% had more than 20 cells participating. Thus, collective modes had a broad distribution of overall firing, and there was only a small fraction of modes with more than 20 out 152 cells participating. In contrast, we can see that the average number of modes containing a given cell had a roughly normal distribution around an average of 6. Thus, individual ganglion cells can belong to multiple modes, and this partitioning is relatively even across the different modes.

### The model accurately captures the statistics of spiking patterns

We found that this model closely reproduced the statistics of the data. Model performance was assessed by training on half the data, and evaluating goodness-of-fit on the other half using the log-likelihood measure. The log-likelihood is given by $L=\sum_{w}f_{emp}\left(w\right)\;\ln P_{\text{model}}\left(w\right)$, where *w* indexes each unique binary spike word in the population, *f*_*emp*_ (*w*) is the empirical frequency of that word in the test data, and *P*_*model*_ (*w*) is the probability predicted by the model. For the full HMM, which includes temporal structure, we took *P*_*model*_ (*w*) to be the stationary distribution (see Fig 2). The overall cross-validated likelihood of our model compared favorably to previous state-of-the-art models (Fig 6), including the K-pairwise maximum entropy model of [25], which incorporates constraints on the mean spike rates, pairwise correlations, and spike-count distribution, and the reliable interactions (RI) model [51]. Because our HMM also reproduces some of the temporal structure of the data, we expect that the full likelihood of the HMM to be even better than indicated here.

Our model was as good as the RI model (with a word-count threshold parameter *n*_*RI*_ = 2 counts) in reproducing the probabilities of words appearing at least two times in the data (Fig 6B). We note that our HMM effectively reached the upper bound on possible performance for this metric, because the RI model’s performance on many of these words reached sampling error, by construction. Our model has the advantage over the RI model of being a well-defined probability distribution, whereas the RI model is a non-normalized pseudo-likelihood. For example, many words occurring only once in the data were assigned “probabilities” greater than one by the RI model. The HMM is therefore more amenable to interpretation and statistical sampling for simulation.

As a detailed assessment of goodness-of-fit, we compared our model’s predictions to the actual values (measured from a held-out test set) of many statistical quantities, again using the stationary distribution of the HMM. The low-order moments were well-reproduced (Fig 7A–7C): the coefficient of determination between model-predicted and true pairwise correlation coefficients was *r*^*2*^ = 0.95 for pairwise correlations and 0.80 for triplet correlations. The incorporation of tree-structured correlations into the emission distributions improved these values relative to a model with only independent emission probabilities; in the latter case the pairwise correlations had *r*^*2*^ = 0.92, and the triplet correlations had *r*^*2*^ = 0.74. Moreover, the model accurately predicted probabilities of individual population words (Fig 7D and 7E) and the probability distribution of population spike count, $k=\sum_{i}\sigma_{i}$ (Fig 7F). The latter is a sensitive measure of the degree of high-order correlation [25].

The empirical frequency distribution of population words was highly skewed, and approximated a power law distribution (see Supporting Information File). Indeed, in this particular dataset, words occurring once each accounted for 90% of the unique observed words, and 38% of the probability mass. The model-predicted probability of such low-count words, and, of course, of the many unobserved words, is poorly constrained by the data. The consequent underestimation of such words’ probability (Fig 7E) translated into a slight downward bias in the predicted second- and third-order moments (Fig 7B and 7C). However, there was no explicit constraint on these moments in fitting the model (in contrast to the maximum entropy paradigm [6, 25, 53]) and the slight inaccuracy in their prediction was compensated by good performance on the frequently occurring words (Fig 7D). Furthermore, a parametric bootstrap analysis showed that the degree of mismatch between predicted probability and empirical frequency in the low-count words was largely consistent with sampling error in a highly skewed distribution (Fig 7E, gray band).

### Modes correspond to discriminable clusters in activity space

We have demonstrated that a hidden Markov model, based on the assumption that neural population responses may be grouped into a collection of discrete modes, constituted an excellent statistical description of our data. Nevertheless, the success of the model is not in itself evidence that the population responses form discrete clusters, since our model contains no constraint preventing the recovered modes from overlapping to an arbitrary degree. Moreover, it is not *a priori* obvious that clustering structure, if present, is due to any nontrivial processing within the retina beyond the pattern of correlations induced by overlapping receptive fields.

To check that the modes identified by our model indeed formed discriminable clusters, we applied linear discriminant analysis (LDA). We first identified the set of unique population words that were mapped into each mode by the model (Fig 8A and 8B, *top*). We carried out Fisher’s LDA to project the set of words assigned to each pair of modes onto the one dimension that best separated them (Fig 8A and 8B, *bottom*) [54]. We then measured the discriminability of modes by *d*′ (the distance between mode means along the LDA axis, scaled by the standard deviation) and identified for each mode the distance to its nearest neighbor, i.e. the mode with smallest *d*′. Such neighboring pairs typically involved the activation of non-overlapping subpopulations of cells. Furthermore, the nearest-neighbor *d*′ was at least 1.5 standard deviations for all modes, and above 2 standard deviations for many modes (Fig 8C). The degree of discriminability also increased slightly with *⟨k⟩*. This is the worst case, nearest-neighbor discriminability: *d*′ between arbitrary mode pairs was typically higher: 1.5 − 4.5 standard deviations (Fig 8D). Since the LDA procedure seeks out the one-dimensional projection of a 152-dimensional space that maximizes *d*′, there is some possibility that this high degree of nearest-neighbor discriminability could be obtained by chance. We therefore repeated the same analysis after randomly shuffling the assignment of time bins to modes. These shuffled modes had robustly smaller *d*′ (Fig 8C and 8D).

**Fig 9:**
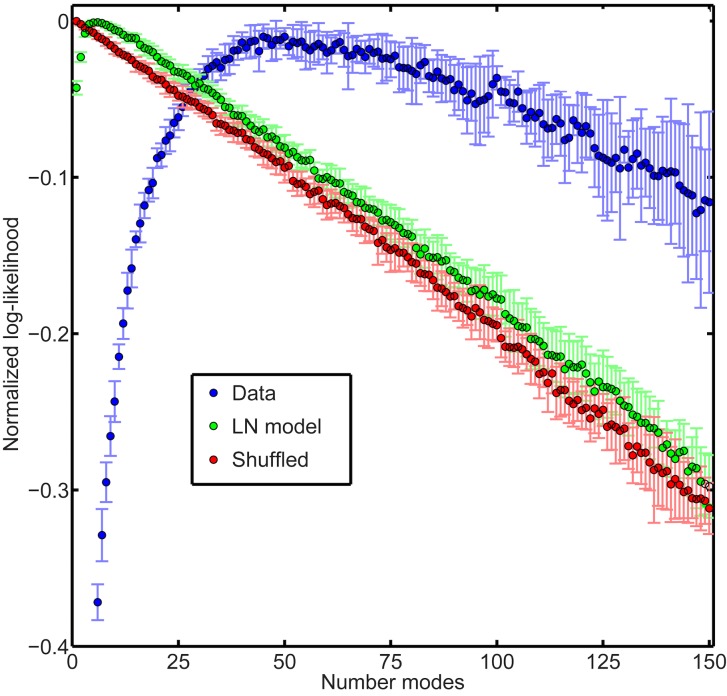
Optimal number of modes in full data compared to simplified controls. Likelihood of the hidden Markov model plotted as a function of the total number of modes. The real data (blue) contained many more modes (50 modes) than either a linear-nonlinear (LN) model fit to the same data (green, 6 modes) or the data with each cell’s spike train randomly shuffled to eliminate correlations (red, 1 mode). All likelihoods were estimated in 10-fold cross-validation. For each fold, we defined a normalized log-likelihood by subtracting the maximum log-likelihood over mode number. These normalized log-likelihoods were then averaged over the 10 folds. Error bars: +/- 1 standard deviation across folds.

We concluded from the above analyses that the modes captured by our model indeed corresponded to discriminable response clusters, especially for patterns with many spikes across the population. We next checked whether the degree of clustering identified by our model was a nontrivial feature of the data by comparing to simplified models. First, the specific patterns of synchronous spiking characterizing our modes may naively be expected to arise from receptive field overlap. We therefore constructed a linear-nonlinear (LN) model of the population that reproduced this effect of common stimulus drive. In order to do so, we analyzed a data set in which the retina was driven by a binary white noise stimulus, so that the receptive fields could be estimated by spike-triggered average analysis [55]. This dataset consisted of 155 ganglion cells. We fit an LN model to each neuron in the recording (Methods) and then simulated data by sampling from the LN model, driven by the actual white noise sequence used in our experiment and assuming conditional independence across neurons. We then fit our model to both the real data and the simulated LN data, and evaluated the model’s likelihood in 10-fold cross-validation to assess the optimal number of modes (Fig 9). There was a substantial difference between the two models, with 50 modes being selected as optimally describing the real data (Fig 9, blue) but only 6 for the LN data (Fig 9, green).

Finally, we verified that correlations were necessary to give rise to mode structure by randomly shuffling each spike train in the data in order to eliminate all correlation between all cells. The resulting shuffled data was best fit by just one mode (Fig 9, red), which is unsurprising since our model with one mode should, in principle, capture uncorrelated data exactly. However, this analysis does demonstrate that the mode structure is a specific consequence of correlated spiking among cells, and not a statistical artifact of sparse firing.

### Modes encode distinct stimulus features

**Fig 10:**
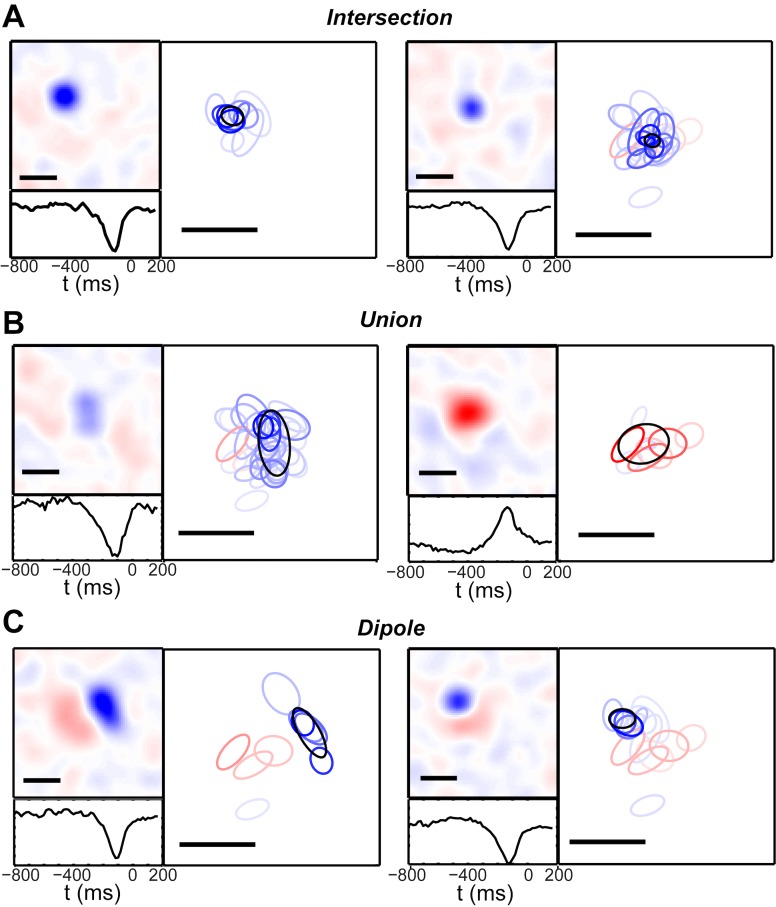
Mode receptive fields. Example receptive fields of modes, measured as the mode-triggered stimulus average during a white noise checkerboard stimulus. In each panel, the left sub-panel exhibits the mode’s spatial receptive field (*top*) and temporal profile (*bottom*). Spatial receptive fields have been smoothed with a Gaussian kernel (1 check standard deviation) and interpolated by a factor of 4. The right sub-panel shows a 1-standard deviation contour of the best Gaussian fit to the mode receptive field (black ellipse) compared to the same fit to individual cells’ spike-triggered average receptive field (red: ON-type cells, blue: OFF-type cells). The saturation of the ellipse’s color represents the cell’s mode-dependent spiking probability *m*_*iα*_. Scale bars = 5 checks. We observed three qualitative categories of mode receptive field: (A) *Intersection*. These had highly localized, compact, and symmetrical receptive fields located at the intersection of many single-cell fields. Half the modes were of the intersection type. (B) *Union*. These were larger and more elongated, were spanned by several individual receptive fields, and occurred as both OFF-type (*left*) and ON-type (*right*). (C) *Dipole*. These received a strong contribution from both ON- and OFF-cells, with the two populations forming separate clusters with low overlap. The overall mode receptive field therefore consisted of an oriented pair of ON- and OFF-subfields.

Our model was fit in an unsupervised way, without explicitly accounting for the stimulus. Nevertheless, the time-varying mode sequence “drives” spiking responses in a manner analogous to the unknown stimulus. It is thus natural to ask whether the modes have any meaningful relationship to the stimulus, and, if so, which features of the visual input are represented. We trained the model on the white-noise checkerboard data introduced above, which was well described by 50 modes. We then estimated the receptive fields of all cells by computing the spike-triggered average stimulus [55], and classified cells into ON- and OFF-type. We next evaluated each mode’s receptive field by taking the mode-triggered stimulus average, i.e. by averaging all stimuli preceding a time bin in which the mode was active. We split all receptive fields (cell and modes) into a spatial and temporal part by finding the best-fitting separable approximation, and compared each mode’s spatial receptive field to those of the cells most active within the mode. Next, we fit the best 2D Difference of Gaussian function to the spatial profile of each mode’s receptive field (see Supporting Information File for details). We used the parameters of these fitting functions to divide the mode receptive fields into four qualitative types: 1) intersection, 2) union, 3) oriented dipole, and 4) independent (Fig 10).

Half the modes resembled the *intersection* of many individual ganglion cells’ receptive fields (24 modes; Fig 10A). These modes had small, compact fields, often concentrated over only 1−4 checks. The receptive fields of the cells with highest mode-dependent firing probability *m*_*iα*_ tended to be larger than the receptive field of the mode itself, and overlapped extensively. This arrangement represents a straightforward mechanism by which the retinal population code can achieve greater spatial acuity than individual cells, by encoding highly localized stimuli through the simultaneous spiking of several cells each containing the stimulus in their receptive field. The intersection-type modes predominantly had an OFF response, and the cells overlapping the center of these were OFF-cells. This may reflect the high asymmetry of OFF- to ON-cells in our recording (122 OFF cells and 33 ON cells), which is characteristic for the tiger salamander retina [30].

Similar features were found to be encoded by the simultaneous spiking patterns of groups of 2–4 retinal ganglion cells [46]. We view our finding of *intersection*-type receptive fields as an extension of these previous results to the case of ~150 cells. However, we also find other qualitatively different spatial features not reported in [46] (see below). In addition, the approach of studying the features encoded by specific, multi-neuronal spiking patterns [46, 47] suffers from the difficulty that combinations of spiking and silence among increasingly many neurons become prohibitively rare to observe. In contrast, our current approach maps many neural activity patterns onto the same collective mode, and consequently our collective modes were all observed with significant frequency (Fig 3A). Thus, our current approach generalizes better to large neural populations.

Other modes had extended receptive fields which were larger than many of the underlying individual cells’ receptive fields. We call these *union*-type modes, and they occurred with both OFF- and ON-polarity (8 modes, 6-OFF type, 2 ON-type; Fig 10B). Extracting such population responses could be useful for detecting complex spatiotemporal stimulus features that require pooling over several spatial subunits. These modes might also embody a form of position invariance for the occurrence of a similar stimulus feature over a wider area.

We quantified the size of *intersection* and *union* mode receptive fields by examining the receptive field radius, defined as the semi-major axis length of the 95% confidence ellipse of the best-fit 2D single Gaussian (see Supporting Information File). The median radius of the *union* modes’ spatial receptive fields was 165 μm, whereas it was 121 μm for *intersection* modes, and 138 μm for *independent* modes (see below). For cells, the median radius was 143 μm. We compared modes’ receptive field radius to the individual cells contributing to the mode by computing an average cell radius, which we denote $r_{\alpha }^{\left(\text{null}\right)}$, for each mode as the weighted average of each cell’s receptive field radius, weighted by the cell’s mode-dependent firing probability (see Supporting Information File). For the union-type modes, the mode receptive field was 16% larger than the average cell receptive field, as a median value, and intersection-type modes were 19% smaller than cells. Both of these differences were statistically significant (p < 0.01, Wilcoxon signed-rank test).

Intriguingly, several modes had *oriented dipole*-type receptive fields (3 modes; Fig 10C). In these modes, OFF-cells and ON-cells both had high mode-dependent spiking probability. The receptive fields of these OFF and ON subpopulations, however, formed distinct spatial regions with minimal overlap. This arrangement resulted in a mode receptive field that had separate ON- and OFF-polarity subfields. These modes are therefore well-suited to detecting an oriented contrast edge. Interestingly, this orientation-selective receptive field is reminiscent of a V1 simple cell [56]. However, we note with caution that the amphibian pallium has been poorly studied, and the responses of tectal neurons–the predominant downstream targets of the retina–have not traditionally been reported to be tuned to static, oriented stimuli [57] (but see [58] for a recent demonstration of orientation selectivity in the mouse superior colliculus). Nevertheless, our results demonstrate that important visual primitives emerge as statistically robust features of the retinal population code. We find this result particularly intriguing, because the stimulus ensemble was spatiotemporal white noise–i.e., having no spatial features with orientation that were statistically over-represented. This suggests that retinal circuitry itself may be biased to extract oriented spatial features from the visual scene.

Lastly, several of the modes had receptive fields that were not statistically different than expected from their constituent ganglion cells (14 modes, not shown in Fig 10; see Supporting Information File for more details). In addition, 1 of our 50 modes had a spatial profile that was too noisy to analyze further.

The temporal profiles of the mode-triggered averages qualitatively resembled those of individual cells, with most having a monophasic and some a biphasic time course. Mode temporal profiles were slower than those of individual cells, which is to be expected due to the autocorrelation in the mode response.

### Collective modes are robust to noise

The results reported in the previous section imply that each mode corresponds to a discriminable cluster of response words. Noise in the retinal response will produce a number of distinct words, all evoked by the same input stimulus. If distinct words, related by noise, preferentially fall within one mode’s cluster, then the mode identity will be robust to noise–i.e., the mode representation would constitute a form of error-correcting code. To test whether this was the case, we evaluated the reproducibility of modes across stimulus repeats.

We evaluated the model’s fit to several different stimulus classes featuring identical, repeated segments: a natural movie (170 cells, 100 modes, 73 repeats), a dark bar extended across the recording area, randomly moving in the transverse direction (140 cells, 30 modes, 108 repeats), and the white noise checkerboard stimulus analyzed above (155 cells, 50 modes, 69 repeats). All three stimuli featured a long period of non-repeated stimulation interspersed with the shorter repeats. We fit the model to the non-repeated data and evaluated reproducibility on the repeated portion.

**Fig 11:**
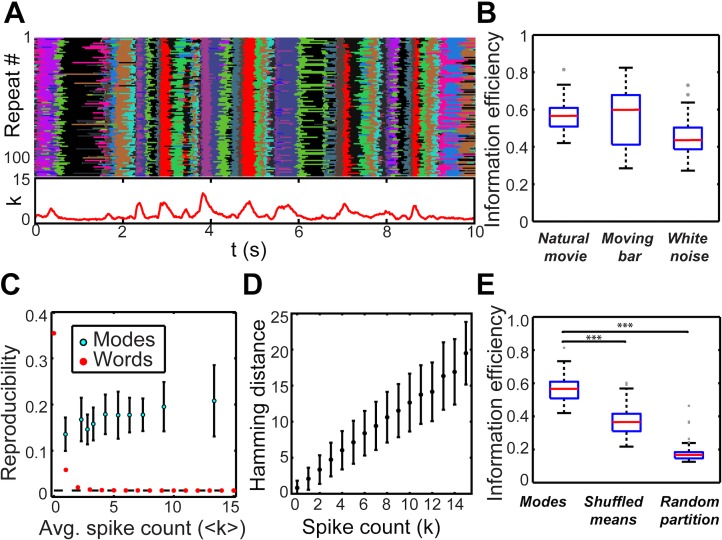
Mode reproducibility across stimulus repeats. (A) *Top*: Mode activation over 108 identical 10 sec repeats of a moving bar stimulus. This dataset included 140 cells and 30 modes. Each color corresponds to a distinct mode; black is the silent mode. *Bottom*: Population spike count averaged over the repeats. Note that reproducibility was generally high, dropping for low *k* and during transitions between modes. (B) Information efficiency of mode activation across repeats of the same stimulus (Information efficiency = mutual information about stimulus / mode output entropy; see Methods), for three distinct stimulus classes. Box plots depict the distribution of this quantity over all modes. Red line = median; blue box = 25% (lower) / 75% (upper) percentiles; black lines = minimum (lower) and maximum (upper), excluding outliers; gray points = outliers. *Natural movie*: 170 cells, 100 modes, 73 repeats. *Moving bar*: same data as (A); 140 cells, 30 modes, 108 repeats. *White noise* (used in Fig 10): 155 cells, 50 modes, 69 repeats. (C) Reproducibility of modes or words across stimulus repeats, defined as the fraction of repeats (out of 73) on which the mode or word occurred within 80 ms of an occurrence at a different repeat, averaged over all times when the mode or word occurred at least once. Reproducibility of the mode response increased slightly with activity level, while that of words dropped to the minimal possible reproducibility (dashed line). For modes, data points were averaged over bins; error bars: ±1 standard deviation. For words, all words sharing the given *k* value were averaged. (D) Hamming distance between pairs of words occurring at the same time across identical stimulus repeats, averaged over pairs. Error bars: ±1 standard deviation. (E) Box plot of mode information efficiency compared to randomized controls. The true modes were significantly more reproducible than either control (***: p < 0.001; Mann-Whitney U test).

The same modes were frequently activated on different stimulus repeats, with especially robust reproducibility apparent at high population activity level *k*. An example portion of the bar stimulus is shown in Fig 11A, where each color indicates a mapping of the neural activity pattern onto the same mode. Generally, mode reproducibility tended to degrade during (i) periods of quiescence (time bins with no more than two cells spiking), and (ii) transitions between modes. Both results are expected *a priori*. During periods of quiescence, too few cells are spiking to embody much robustness to noise, and during transitions between modes, there must always be one point in time in which the likelihood of the earlier and later modes is exactly equal.

To quantity the overall degree of reliability of modes, we generated a binary vector for each mode, which was 1 when the mode was active at a given time bin and 0 otherwise. We then calculated, for each mode, the fraction of the mode’s entropy that was informative about the stimulus (see Methods). This “information efficiency” measure had a median of 0.57 for the natural movie and 0.60 for the bar stimulus; moreover, it was above 0.80 for some modes. Information efficiency was lower for the less-structured white noise, with a median of 0.44, but still comparable to the other stimuli for many modes (Fig 11B). Moreover, the information efficiency of modes exceeded that of individual cells. For the natural movie, the median efficiency of single cells was 0.41 (c.f. 0.57 for modes). The most efficient single cell reached 0.68, while the best mode had an efficiency of 0.81.

Since the natural movie stimulus is most representative of the retinal input under ecological conditions, and it evoked the largest number of modes, we focused further analysis on this dataset. We sought to compare the reproducibility of individual population spiking words to the reproducibility of modes. Since most individual words occur very rarely, and not at all on some repeats, the above information measure is poorly estimated for words. We instead quantified the reproducibility of words (modes) as the fraction of repeats on which a word (mode) was active at a particular time bin, given that the same word (mode) occurred within 80 ms on a different repeat. Individual words were highly variable, with reproducibility decreasing with *k* and dropping to its minimal possible level by *k* = 5 (Fig 11C). In fact, high-activity words often occurred only once each in the entire dataset. Nevertheless, mode reproducibility was substantially higher and increased slightly with activity level (Fig 11C).

We also quantified noise in the underlying population response by the average Hamming distance between pairs of words occurring at the same time bin within the stimulus, but on different repeats. By this measure as well, population responses were quite noisy, with Hamming distance exceeding spike count *k* and increasing with activity level (Fig 11D). This result is consistent with the variability in the spike count of individual single ganglion cells [59] and provides additional insight into the low reproducibility of high-activity words.

Certain trivial effects may explain the high level of mode reproducibility. We sought to control for these by carrying out two manipulations comparing the mode assignment produced by our model to randomized modes preserving only limited structure (Fig 11E). Details of the construction of both controls are reported in Methods. First, there is a “similarity effect”: the model assigns to the same mode words which are similar to one another or that occur close in time (due to the temporal correlation incorporated into the model). This grouping by similarity and temporal proximity may automatically reduce noise. We constructed a control which randomized the location of each mode in response space–its mean–while approximately preserving its entropy, as well as preserving the overall form of the HMM (including temporal correlations). To ensure that the temporal correlations were optimal relative to the newly constructed modes, we re-fit the full HMM while fixing the randomized mode means. We will refer to this construction as the “shuffled means” manipulation. It groups words by similarity to the randomized means and by temporal proximity induced by the learned temporal correlations, and thereby reproduces the similarity effect. This control produced modes that were significantly less informative than the true modes identified by our model (Fig 11E), with a median information efficiency of 0.37. The fact that this randomized control still carried some information likely reflects the underlying reproducibility of single cells, since some of the random modes ended up being highly selective for certain individual cells. Indeed, the median single-cell efficiency was 0.41, quite close to the value for the shuffled means control.

Another possible explanation for the high degree of reproducibility is that the mode representation compresses tens-of-thousands of words into a much smaller number of modes. This large reduction of entropy may be expected to enhance reproducibility by chance. To control for this “compression effect”, we constructed modes in which each word was assigned to a random, unique mode, while matching the overall number and probability distribution of modes to that in the full model. We refer to this control as the “random partition”. We found that modes defined by random partition had very poor information efficiency, with a median of 0.17 (Fig 11E). The random partition modes in fact had a reproducibility near chance, here defined by randomly permuting the time bins within each stimulus repeat before calculating information efficiency on the original basins. This chance value had a median of 0.14. The only randomized modes that were comparably informative to the true modes were those that contained the all-silent state and other very low spike count words.

There is also a “word reliability effect”: although most individual words had extremely low reproducibility (Fig 11D), some were more reliable across repeats (in particular, the all-silent state–which is also overwhelmingly the most frequently occurring word). Therefore, it is conceivable that modes automatically inherit this reproducibility, even without detecting any special structure. Both randomized manipulations control for this reliability effect.

Together, the poor information efficiency of both of these controls (compared with that of the true modes) suggests that there is an underlying structure to the data that is captured by the full model, but not by arbitrary mappings of activity patterns onto modes. Thus, collective modes form a representation of the visual stimulus that is highly robust to noise in the activity of individual neurons.

## Discussion

We have demonstrated that the spike trains emitted by the ganglion cells composing a densely covered patch of retina are statistically structured into a sequence of discrete collective modes. This decomposition provided an accurate model of the retinal output under a variety of stimuli, including natural movies. Different modes were readily discriminated from each other, especially at high activity levels (Fig 8). These modes carried meaningful information about the stimulus, as their activation was correlated with specific stimulus features not present in individual cells (Fig 10). Finally, we found that modes were highly robust to noise in comparison to individual words: the response fluctuations that occur over identical presentations of the stimulus preferentially mapped words onto the same mode (Fig 11).

### Adopting a neural coding approach

There are two distinct and complementary ways to create a probabilistic model of the retinal output (or, indeed, of any sensory system). One approach attempts to explicitly capture the dependence of neural responses on the stimulus. Models of this class include standard linear-nonlinear (LN) models, generalized linear models (GLMs) [26] [60], and stimulus-dependent maximum entropy models [61]. These models, which we will call “encoding” models, have two primary advantages: they may highlight the circuit-level computation by which the retina transforms its input, and they provide a principled basis for deriving decoders that transform the retinal output into a reconstruction of the stimulus [26, 62]. Mathematically, all such models are parameterizations of the conditional distribution of responses on stimulus, *P*({*σ*_*i*_(*t*)}|*s*(*t*)), where *s(t)* denotes the stimulus. Many versions of such models also have the benefit that they can be applied to arbitrary patterns of light, putting them at the highest level of generality.

The drawback of this approach is that these models do not correctly predict ganglion cell spike trains in many, if not most, visual conditions. To give a few examples: 1) LN models (and their generalizations including ganglion cell gain control) do a good job predicting the firing rate for objects moving at constant velocity [63, 64], but fail for discontinuities of motion [65–67] or for wide field motion [68, 69]; 2) LN models (and their generalizations including refractory periods) do a good job for random flicker that is spatially uniform or nearly uniform across the receptive field center [26, 70, 71], yet fail for random flicker with small checkers [72]; 3) even the primate midget and parasol cells, which seem more closely linear than most other ganglion cell types, require models with multiple gain control stages to predict the firing rate for temporal-chromatic natural movies [73].

The picture that emerges from these studies is that there are multiple sources of non-linearity within the retinal circuit that are significant in some (but not all) visual conditions and that endow ganglion cells with light responses qualitatively different from the LN model. Examples include bipolar cells with rectification and synaptic depression in their axon terminal [74–76] as well as different types of amacrine cells that veto ganglion cell response when specific visual features are present [68, 77–80]. Given the extreme diversity of amacrine cell types [81] as well as the fact that they can individually remold each bipolar-to-ganglion cell transfer function [82], the prospect for deriving an input-output model that will succeed over the entire range of natural visual conditions currently seems quite remote.

An alternative approach–the one adopted in the present work–is to directly model the structure of retinal responses, without reference to the stimulus. Mathematically, these models represent the distribution *P*({*σ*_*i*_(*t*)}). Models of this form, which we call “activity” models, have their own advantages, complementary to those of encoding (stimulus-dependent) models. Most importantly, they correspond to the problem faced by the rest of the brain, which lacks direct access to either the stimulus or the internal structure of the retina. These models, rather than highlighting the circuit-level computation, instead emphasize the structure of the neural code produced by the system.

Such activity models address the question of how the information conveyed to the rest of the brain is *formatted* by the retina, and therefore how it could be most naturally interpreted. Note that this question is quite distinct from the notion of stimulus decoding–in our view, the goal of the visual system is decidedly *not* to reconstruct the exact light pattern comprising the stimulus, which anyway already exists in the photoreceptors. Rather, the goal is to amplify behaviorally relevant stimulus features while suppressing irrelevant ones–e.g., the operation of recognizing the same object in the environment under different viewing conditions [83]–which is ultimately a complex function of information encoded at the photoreceptor level. Activity models provide neuroscientists with data-driven hypotheses of what these features may be. Our results reported in the present paper suggest that the collective modes are one example of a relevant signal emphasized at the retinal ganglion cell level; there could be others as well.

One advantage of activity models is that they can be formulated in circumstances in which there exists no satisfactory encoding model, such as under natural movie stimulation. The method is simply to measure simultaneously the activity states of the neural population over a stimulus ensemble, as is possible with techniques like multi-electrode recording or optical imaging. In taking this approach, we were able to identify many more collective modes in response to checkerboard flicker than could be identified with matched LN models of each neuron (Fig 9). This result demonstrates the benefit of direct experimental measurement of neural activity over inferences from encoding models, at least in the context of our study.

A consequent obvious disadvantage of the approach based on activity models is that the distribution of states, *P*({*σ*_*i*_(*t*)}), will depend strongly on the choice of stimulus ensemble. However, it should be pointed out that encoding models also suffer implicitly from this difficulty, as the form of model that succeeds for one stimulus ensemble may not succeed in others. This limitation is a consequence of the aforementioned circuit complexity arising from gain control, amacrine cell network interactions, and other effects. Finally, we note that even a perfect encoding model–which would necessarily be far more complex and sophisticated than the present state-of-the-art models–would require an analysis along the lines of the present work in order to address questions at the level of the output neural code that we have studied here.

Our modeling work suggests that neural circuits downstream of the retina can extract meaningful information about the visual stimulus merely by clustering retinal activity patterns into a sequence of collective modes. Our model enabled us to extract population modes that were informative about the stimulus and robust to noise. Since the modes were defined without any information about the stimulus (i.e., its repeat structure), the reproducibility results of Fig 11 are highly nontrivial. Furthermore, these modes often encoded features of the stimulus that were not represented by any individual neuron.

### Properties of the collective modes

How much information is carried by the sequence of modes? For our non-repeated natural movie stimulus, with 70 modes, the modes carry 2.0 bits/s per neuron of entropy. This is about 25% of the maximum-entropy upper bound on total response entropy estimated in [25] for a separate dataset of salamander ganglion cells responding to a natural movie. There are three possible explanations for this gap, which may be explored in future work. First, much of the population entropy is noise: estimating noise entropy for the dataset used in [25], we find that almost 60% of the population entropy is noise entropy (this is an upper bound, assuming neurons are conditionally independent given the stimulus). Since we found that modes are less noisy than population words, we expect a smaller fraction of the mode entropy to be noise.

Second, the modes as we have defined them may only represent one channel of meaningful stimulus information. Further information may be represented as substructure of the modes, or as a distinct partitioning of the population activity. Such a multiplexed population code would then allow different downstream targets to readily extract different information streams from the same input. For example, given that we find substantial variation of population spike count within each mode, one hypothesis may be that mode identity represents a particular stimulus feature while population spike count encodes the contrast or intensity of that feature. Other examples of such multiplexed encoding are known to exist. In the retina, contrast adaptation leads to a multiplexed neural code: the visual information encoded by individual spikes is roughly invariant to the contrast, while the total firing rate encodes the absolute contrast on a longer time scale of ~10 sec [84, 85]. In the visual cortex, information about different aspects of the stimulus–such as the spatial phase, contrast, and orientation of gratings–is represented at different time scales in the same cortical spike trains [86, 87].

Finally, we may be vastly underestimating the total number of modes, as we are limited by finite sampling. With greater sampling–note that our recording times of ∼ 1 hr are orders of magnitude lower than what’s available to the organism under natural conditions–many more rare words would be expected to occur. These new words would form new modes, and the entropy of the probability distribution over modes would increase. In contrast, the aforementioned maximum entropy bound should be fairly stable against improved sampling, since it’s based on a maximum entropy model constrained on a set of consistent statistics (indeed, as more structure is revealed, the true population entropy may decrease further below the maximum entropy bound). Likewise, sampling a larger fraction of the cells overlapping the patch of visual field would likely yield a larger number of more readily distinguished modes. In contrast, the entropy per cell reported in [25] continues to decrease with neuron number, apparently not saturating until 200 − 300 neurons. Therefore, we expect the fraction of total entropy captured by the modes to increase with sampling in both time and cells, provided that the observations we have made about the response distribution are generalizable to unobserved data.

### Other activity models

One approach to identifying error-robust sets of codewords was suggested by the application of maximum entropy (MaxEnt) models that constrained the pairwise correlations among retinal ganglion cells [6]. The pairwise interactions learned by fitting these models typically feature both positive and negative interaction strengths. In models of physical magnetic systems, such disordered interactions are known to lead to a probability distribution featuring many local peaks [88]. “Error-corrected” codewords have been identified in retinal data as local maxima of the probability landscape defined by MaxEnt models, and indeed were shown to encode reliable information [25]. Fitting maximum entropy models is computationally demanding, especially for datasets from a large number of cells. Moreover, the definition of local probability maxima depends on an arbitrary choice of metric distance between population states–such as the Hamming distance–which may not weigh cells in a way that facilitates optimal discrimination. We therefore adopted a more direct approach to extracting the robust collective representations of the retinal population code, by exploring a model of population neural activity that explicitly incorporates dependence of the observed spike patterns on a hidden variable identifying the population mode. The coding symbols identified in [25] persisted with an average timescale of 48 ms, consistent with the dwell time of modes that we discovered by our methods. Therefore, we predict a close relationship between the two constructions.

Latent variable models have been successfully applied to other neural systems; several related approaches and findings have recently been reviewed [89]. Dimensionality reduction methods applicable to single-trial spiking data, the paradigm adopted in our work, include continuous state-space models in addition to HMMs. Hidden Markov models have been employed to capture population spiking in a variety of cortical structures, including frontal [90–92], premotor [93], gustatory [94], and somatosensory areas [91]. A similar latent-variable-based approach, restricted Boltzmann machines, were applied to visual cortex in [95]. Relatedly, a clustering algorithm was employed to show that auditory cortical dynamics were organized into transitions between discrete states [96]. Our methodology extends the prior applications of HMM, which assumed conditional independence of different neurons within each hidden state, by introducing correlations into the emission distribution in a way that enables efficient inference. Moreover, that a discrete state-space model such as HMM should apply to the retina, as we have demonstrated, is perhaps more surprising than in cortex, where such a discrete structure may be expected as a consequence of recurrent attractor dynamics or global brain state fluctuations. In the present work, we have argued that such discreteness of the population response is desirable in early sensory stages for functional reasons, facilitating error-robust encoding of information.

An important question in extending the present model to larger neural populations is how the number of modes scales with the number of neurons. For the dense, local patch of 100–200 retinal ganglion cells sampled in the experiments analyzed in this paper, we found that the optimal number of modes was less than the number of neurons. However, we expect this result to be a consequence of the high degree of redundancy among these cells, due to their mutual receptive field overlap. If cells are not so highly correlated, the number of modes may scale faster. For example, for our model to describe two completely non-overlapping and statistically independent retinal patches would require a number of modes equal to the *product* of the number of modes in each patch. To describe such data, future work could explore a hierarchical model tying together several HMMs into deeper layers, modeling structure at a variety of scales. More generally, the appropriate latent variable model for a given neural population will depend on the balance between redundancy and synergy. We expect our results to apply to predominantly redundant populations elsewhere in the brain.

### Population neural codes beyond the retina

The general problem faced by the retina is to extract relevant features from a complex, high-dimensional input, and then to package those into output spike trains in a way that facilitates downstream recognition of the relevant features while suppressing irrelevant features (e.g., noise). This way of thinking about neural computation clearly generalizes beyond the retina, applying broadly across the nervous system. The techniques introduced in the present work allow such hidden relevant features to be explicitly inferred from output spike trains alone, and our application of these ideas to the retina forms a simple test case in which the input–the visual stimulus–is well understood.

Future work could apply our model to analyze the collective mode structure of more complex, higher-order, brain areas where the relevant features of the input are unknown. For example, in a study that recorded from large populations of neurons in the inferotemporal cortex while the monkey was viewing different categories of objects, the activity states of the neural population clustered into categories that matched the object categories [97]. In both this study and in ours, the clustering of activity states was only observed in large populations. Measurement at the scale of hundreds of neurons may reveal this structure in other brain areas as well.

In cortical areas involved in higher-level sensory perception or decision making, the partition of the input into “relevant” and “irrelevant” features could be dynamic, depending on behavioral demands. It would be quite interesting to apply our model to such areas to see how the mode structure changes with attentional state or task structure. Our approach also reduces complex population activity to a single, scalar, codeword–the mode identity. In brain areas where individual neurons demonstrate difficult-to-interpret mixed selectivity, it may be the case that the collective representation is more explicit than that of any single neuron [98]. Our model provides a straightforward method to test similar hypotheses and to explicitly identify the relevant population codewords in spiking neural populations across the nervous system.

## Methods

### Ethics statement

This study was performed in strict accordance with the recommendations in the Guide for the Care and Use of Laboratory Animals of the National Institutes of Health. The protocol was approved by the Institutional Animal Care and Use Committee (IACUC) of Princeton University (Protocol Number: 1828).

### Experimental procedures

We recorded responses from larval tiger salamander retinal ganglion cells using a custom 252-electrode multi-electrode array (MEA). For all but the natural movie experiments, eyes were removed in darkness and the retina separated from the pigment epithelium. The natural movie experiments were conducted with the pigment epithelium intact and surgery performed in dim light. The isolated retina was then placed onto the array and superfused with oxygenated Ringer’s medium at room temperature. Offline spike sorting was performed with custom software. Detailed recording and analysis methods, including the spike-sorting algorithm, are described in [1].

### Visual stimulation

Stimuli were projected onto the array from a CRT monitor at 60 Hz. We presented three stimulus classes, in different experiments: a natural movie, a binary white noise checkerboard, and a diffusively moving dark bar. The natural movie consisted of a 7–minute gray scale recording of leaves and branches blowing in the wind. We conducted natural movie experiments with two different designs: in the first, the movie was looped with a different pixel centered on the recording area for each repeat. Since the patch of retina recorded by the MEA subtended only a small portion of the stimulus, we were able to construct this stimulus in such a way that the retinal input was effectively non-repeated over the full recording session. In a second design, we generated a similar “non-repeated” movie, but alternated every 60 sec of non-repeated stimulation with an identical 60 sec clip. The binary white noise stimulus consisted of a 40 x 40 flickering checkerboard, randomly updated every frame. Each checker had a size of 55 x 55 μm when projected onto the retina. The drifting bar’s motion was a random walk subject to a restoring force to keep it, on average, centered on the recording area [99]. Both the checkerboard and drifting bar designs consisted of a long period of non-repeated stimulation interspersed with repeated segments at randomly-chosen time intervals (repeat duration = 60 sec for the bar, 30 sec for the checkerboard).

### Model specification

We binned spike trains into 20 ms time bins, producing a sequence of binary spike words *σ*_*i*_(*t*), where *i* = 1…*N* labels the neuron identity and *t* the time bin. We set *σ*_*i*_(*t*) = 1 whenever at least one spike occurred within bin *t*, and 0 otherwise. Because our hidden Markov model (HMM) incorporates temporal correlations between adjacent time bins [50], we fit the parameters of this model to the *entire sequence* of binary spike words, which we denote by ({*σ*_*i*_(0),…,*σ*_*i*_(*T*)}) (see Fig 2):

$$P_{HMM}\left(\left\{\sigma_{i}\left(0\right),\ldots ,\sigma_{i}\left(T\right)\right\}\right)=\sum_{\alpha \left(t\right)}^{\text{mode sequences}}\prod_{t=1}^{T}Q_{\alpha \left(t\right)}^{\text{tree}}\left(\left\{\sigma_{i}\left(t\right)\right\}\right)P\left(\alpha \left(t\right)|\alpha \left(t-1\right)\right),$$

where the summation is over all possible sequences of modes from time 0 to *T*. Because there are *M* possible modes in each time bin, this sum involves a total of *M*^*T*+1^ terms. Here, *α*(*t*) and {*σ*_*i*_(*t*)} denote the mode index and binary spike word in a single time bin, *t*, respectively. The transition matrix was assumed to be stationary, i.e. independent of *t*: *P*(*α*(*t*)|*α*(*t*−1)) ≡ *P*(*α*|*β*). The emission probabilities $Q_{\alpha }^{\text{tree}}\left(\left\{\sigma_{i}\right\}\right)$ were defined by constraining joint probabilities between certain neuron pairs as follows:

$$Q_{\alpha }^{\text{tree}}\left(\left\{\sigma_{i}\right\}\right)=\prod_{i}^{\text{cells}}p_{\alpha }\left(\sigma_{i}\right)\prod_{\langle i,j\rangle }^{\text{edges}}\frac{p_{\alpha }\left(\sigma_{i},\sigma_{j}\right)}{p_{\alpha }\left(\sigma_{i}\right)p_{\alpha }\left(\sigma_{j}\right)},$$

where the pairs indexing the second product, ⟨*i*,*j*⟩, are chosen to form a tree (Fig 2B). This tree constraint is necessary for the factorized form to give a correctly normalized probability distribution; the choice of tree topology, however, is *a priori* arbitrary and was learned during the fitting step. The motivation behind this choice was that these interaction terms help describe correlations within the neural population, yet have a form with parameters that can be readily fit to data. In the statistics literature, this emission distribution is known as the Chow-Liu tree [100]. In our model, the tree topology may differ for each mode α.

The parameters of the full model are therefore: *M*, the number of modes; the *M* × *M* transition matrix *P(α | β)*; the choice of tree topology for each mode; and the collection of joint probabilities on each mode’s tree, *p*_*α*_(*σ*_*i*_,*σ*_*j*_). The latter may be parameterized by a mode-dependent spiking probability for each cell, *m*_*iα*_ = *p*_*α*_(*σ*_*i*_ = 1), and a pairwise correlation for each tree edge, *C*_*ijα*_ = *p*_*α*_(*σ*_*i*_ = 1,*σ*_*j*_ = 1), giving *M* (2*N* − 1) parameters describing the emission distributions. Finally, we introduced a single regularization parameter η, described below.

After fitting the full model, we inferred the mode that was active at each time bin of the data by maximizing the posterior probability of the mode sequence, conditioned on all the observed spike train data from the beginning of the experiment up until time *t*: α*(*t*) = argmax *P*_*HMM*_(*α*(*t*)|({*σ*_*i*_(0),…,*σ*_*i*_(*T*)})). This maximization was implemented by the Viterbi algorithm [101].

For some purposes, we wanted to reduce the full HMM to a static (time-independent) probability distribution, which describes the probability of occurrence of a single binary spike word. This static probability distribution had a mixture form (see Fig 2C):

$$P_{mix}\left(\left\{\sigma_{i}\right\}\right)=\sum_{\alpha }^{\text{modes}}w_{\alpha }\;Q_{\alpha }\left(\left\{\sigma_{i}\right\}\right).$$

where we used the set of tree emission distributions fit for the full HMM along with mode-dependent weights, {w_α_}, defined by the detailed balance equation:

$$w_{\alpha }=\sum_{\beta }^{\text{modes}}p\left(\alpha |\beta \right)w_{\beta }.$$

### Model fitting

For fixed *M*, we inferred the parameters by maximum likelihood, using the Baum-Welch algorithm with an *M*-step modified to accommodate the specified form of *Q*_*α*_ [50]. Full details are reported in the Supplement.

To mitigate overfitting, we introduced a regularization parameter *η* ∈ [0,1] (see Supporting Information File). The above maximum likelihood fitting was carried out for fixed *M* and *η* Increasing *M* and decreasing *η* both increase the complexity of the model, and lead to a strictly improved likelihood on the training data. To choose these parameters we carried out an *n*-fold cross-validation procedure; generating *n* non-overlapping splits of the data into training and test sets. *M* was then chosen to maximize the test set likelihood, averaged over the *n* folds (Fig 2D). In practice, we chose *n* = 2.

Our results were little affected by the precise value of *η*, provided it was small but nonzero. We used *η* = 0.002 throughout the present work.

### Entropy of emission distributions

To calculate the entropy of the emission distributions plotted in Fig 2, we used an analytical formula obtainable from the above-factorized probability distribution:

$$S_{\alpha }=-\sum_{i}^{cells}\sum_{\sigma_{i}}^{\left[0,1\right]}p_{\alpha }\left(\sigma_{i}\right)\log_{2}p_{\alpha }\left(\sigma_{i}\right)-\sum_{\langle ij\rangle }^{edges}\sum_{\sigma_{i},\sigma_{j}}^{\left[0,1\right]}p_{\alpha }\left(\sigma_{i},\sigma_{j}\right)\log_{2}\frac{p_{\alpha }\left(\sigma_{i},\sigma_{j}\right)}{p_{\alpha }\left(\sigma_{i}\right)p_{\alpha }\left(\sigma_{j}\right)}.$$

After fitting the full model (see above), we were able to obtain all parameters necessary to evaluate this equation exactly.

To calculate the maximum entropy possible for an emission distribution with a given average spike count, <*k*>, we started by observing that the maximum entropy would be obtained when all possible states were equally likely. For an integer spike count, this uniform emission distribution is given by:

$$Q_{uniform}^{k}\left(\left\{\sigma \right\}\right)=\frac{1}{C\left(N,k\right)}=\frac{\left(N-k\right)!k!}{N!}.$$

Thus, the maximum entropy is given by:

$$\begin{array}{l} S_{\max}\left(\langle k\rangle \right)=\;-\sum_{\left\{\sigma \right\}}Q_{uniform}^{\langle k\rangle }\left(\left\{\sigma \right\}\right)\log_{2}Q_{uniform}^{\langle k\rangle }\left(\left\{\sigma \right\}\right)\\ \;=\;\log_{2}\frac{\Gamma \left(N+1\right)}{\Gamma \left(N-\langle k\rangle +1\right)\Gamma \left(\langle k\rangle +1\right)} \end{array},$$

where Γ(*N*) is the Gamma distribution.

### Transition entropy

To characterize the statistics of how one mode transitions to another mode in the next time step, we calculated the transition entropy:

$$H_{trans}\left(\beta \right)=-\sum_{\alpha }^{\text{modes}}p\left(\alpha |\beta \right)\log_{2}p\left(\alpha |\beta \right).$$

This quantity has the interpretation that from mode *β*, transitions can be made to roughly $2^{H_{trans}}$ different modes. Because self-transitions dominated, we also calculated the transition entropy using transition probabilities with only off-diagonal components: $\tilde{p}\left(\alpha |\beta \right)=p\left(\alpha |\beta \right)/\left(1-p\left(\alpha |\alpha \right)\right)$ for *α* ≠ *β*.

### Information efficiency

For each mode, we computed *r*(*t*), the fraction of stimulus repeats on which the mode was active at time bin *t*, and its time average $\bar{r}=\frac{1}{T+1}\sum_{t}r\left(t\right)$. We then defined the output entropy of mode occurrence, *S*_*out*_, and its noise entropy, *S*_*noise*_, as follows:

$$S_{out}=-\bar{r}\log_{2}\bar{r}-\left(1-\bar{r}\right)\log_{2}\left(1-\bar{r}\right)$$

$$S_{noise}=-\frac{1}{T+1}\sum_{t=0}^{T}r\left(t\right)\log_{2}r\left(t\right)+\left(1-r\left(t\right)\right)\log_{2}\left(1-r\left(t\right)\right).$$

The information efficiency was defined to be the mutual information divided by the output entropy: (Sout−Snoise)Sout.

### Linear-nonlinear modeling

For each cell individually, we fit a linear-nonlinear (LN) model with a logistic nonlinearity:

$$p_{i}\left[s\left(\bar{x},t\right)\right]=\frac{1}{1+\exp\left(\alpha K_{i}\left(\bar{x},t\right)*s\left(\bar{x},t\right)-\theta \right)},$$

where $p_{i}\left[s\left(\bar{x},t\right)\right]$ is the probability of the cell *i* spiking within time bin *t*, given the stimulus $s\left(\bar{x},t\right)$ in that time bin, and spiking is modeled as independent (Bernoulli) across time bins. The stimulus $s\left(\bar{x},t\right)$ and the linear filter $K_{i}\left(\bar{x},t\right)$ contain both spatial (pixels) and temporal (past time bins) dimensions, with *K*_i_*s denoting summation over the spatial dimensions and convolution over the temporal:

$$K\left(\bar{x},t\right)*s\left(\bar{x},t\right)=\sum_{\bar{x}}^{\text{pixels}}\sum_{\tau }^{\text{time}}K\left(\bar{x},\tau \right)s\left(\bar{x},t-\tau \right),$$

where *τ* indexes time bins and $\bar{x}$ spatial pixels.

To fit the model, we first set the linear filter $K_{i}\left(\bar{x},t\right)$ equal to the spike-triggered average in a binary white-noise checkerboard experiment. In our experiments, the receptive field centers typically occupied a small region of only 2–4 checks across, while the stimulus was 40x40 checks. We therefore set all checks outside of a 7x7 patch centered on the receptive field peak to zero, in order to suppress noise introduced in the spike-triggered average estimate. The remaining two parameters *α* and *θ* were then estimated by numerical maximum likelihood on the model.

### Randomized reproducibility controls

#### Shuffled means

For this control, we omitted the tree-correlations from the HMM emission distributions, instead fitting an HMM with independent emission distributions fully parameterized by the mode-dependent mean vectors *m*_*iα*_. Starting from this model fit to data, we randomly permuted the cell labels of the mean vector *m*_*iα*_, independently for each mode *α*. This generated a set of random mean vectors *m*^*shuff*^. We then partially re-fit the HMM, fixing the mode means at *m*^*shuff*^, while allowing the transition probability matrix to be optimized.

#### Random partition

This control was based on constructing a deterministic, rather than probabilistic, mapping from words to modes. The goal was to partition words into a set of random modes with number and probability distribution matched to that of the real modes. To do this, we first assigned each mode a “capacity” initially equal to its true probability. We then ranked the words in decreasing order of probability. Proceeding in rank order, we mapped each word to a random mode chosen from the set of modes with capacity greater than the word’s probability. After assigning a word to a mode, that mode’s capacity was decremented by the word’s probability before assigning the next word. Eventually, words were encountered with probability greater than any mode’s remaining capacity. In this case, the word was simply assigned to a random mode. Since these words’ probabilities were tiny, the probability distribution of the randomly constructed modes was extremely close to that of the true modes.

## Supporting Information

S1 Text Supporting Information. (PDF)
