# Correction: Error-Robust Modes of the Retinal Population Code

**DOI:** 10.1371/journal.pcbi.1005855

**Published:** 2017-11-10

**Authors:** The PLOS Computational Biology Staff

## Notice of Republication

This article was republished on February 3, 2017, to correct errors in figure dimensions for Figs 2, 3, 7 and 9 that were introduced during the typesetting process. The publisher apologizes for the errors. Please download this article again to view the correct version. The originally published, uncorrected article and the republished, corrected articles are provided here for reference.

## Supporting information

S1 File Originally published, uncorrected article. (PDF)

S2 File Republished, corrected article. (PDF)
