# Supplementary material for: Error-Robust Modes of the Retinal Population Code
Source: PLoS Comput Biol. 2016 Nov 17;12(11):e1005148. doi: 10.1371/journal.pcbi.1005148 (PMC5113862; doi:10.1371/journal.pcbi.1005148)
Supplement: S1 Text — (PDF) [file pcbi.1005148.s001.pdf]

ERROR-ROBUST MODES OF THE RETINAL POPULATION CODE: SUPPLEMENTAL INFORMATION

JASON S. PRENTICE, OLIVIER MARRE, MARK L. IOFFE, ADRIANNA R. LOBACK, GAŠPER TKAČIK, MICHAEL J. BERRY II

Contents

§1. Supplemental Figures 2
§1.a. Supplementary Figure 1. Zipf plot 2
§1.b. Supplementary Figure 2. Relation between number of modes and cells. 3
§2. Supplemental Experimental Procedures 4
§2.a. Fitting a simplified model 4
§2.b. Maximum likelihood estimation of model parameters 5
§2.c. Alternative parameterization of the tree distribution 8
§2.d. L_1 regularization 9
§2.e. Computation of the correlation matrix in the tree model 10
§2.f. Maximizing the posterior over mode sequences 11
§3. Characterizing Mode Spatial Receptive Fields 12
§3.a. Noise & oriented dipole analysis 12
§3.b. Intersection & union analysis for monopole mode RFs 14
§3.c. Classification of intersection, union & independent monopole modes 15
§3.d. Supplemental figures for the mode analyses 16
References 16

§1. Supplemental Figures

§1.a. **Supplementary Figure 1. Zipf plot.**

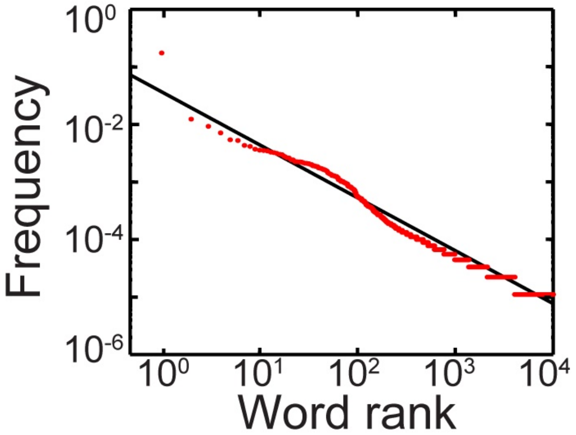

Figure S1. **Zipf plot.** Frequency of individual unique words vs. rank in the non-repeated natural movie experiment; log-log scale. The black line is the power law best fitting the data excluding the silent state and fewer than 3-count words (exponent = −0.99; 95% CI = [−1.0, −0.98]). The data therefore approximates a Zipf law, which is defined by frequency ∝ 1/rank.

§1.b. Supplementary Figure 2. Relation between number of modes and cells.

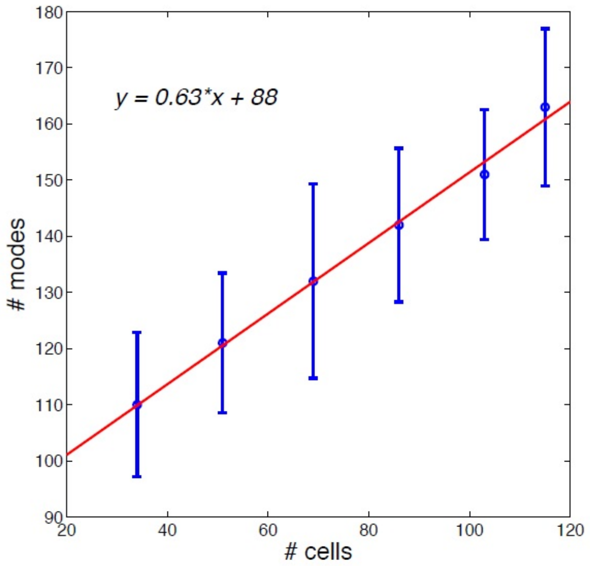

Figure S2. **Number of modes as a function of number of cells.** We generated random subsets of $30, 40, \ldots, 150$ cells such that each subset was contained within the next larger subset (i.e., a random 30-cell subset was chosen, then 10 random additional cells were adjoined to generate the 40-cell subset, and so on). For each subset, we fit the model and identified the optimal number of modes by maximizing the test likelihood in 2-fold cross-validation, as described in the main text. We repeated this procedure 10 times, with different randomly-generated cell subsets. Dots = average number of modes over the 10 repetitions, error bars = ± 1 standard deviation. Line = best linear fit. The scaling of mode number with cell number is approximately linear with a small slope (less than unity).

§2. Supplemental Experimental Procedures

§2.a. **Fitting a simplified model.** Before describing the full methodology of fitting the hidden Markov model described in the main text, we will first build intuition by presenting a simpler, special case of the full model. This model is a mixture model (so time bins are statistically independent, unlike in the hidden Markov model) with independent emission distributions (it lacks the tree-based interneuronal correlations included in the full model). The algorithm for fitting this model is parallel to that for the full model, with some steps simply being more elaborate in the full model. This model is highly computationally efficient to fit, and did a reasonably good job of accurately capturing the statistics of our data (though it was improved upon by the full model reported in the paper).

The independent mixture model assumes that the population responses across time bins $t = 0...T$, each denoted by $\{\sigma_i(t)\}$, are independent across time. Specifically, it formulates that the probability of a single population response is given by:

$$P_{\text{mix}}\big(\{\sigma_i(t)\}\big) = \sum_{\alpha}^{\text{modes}} w_\alpha Q_\alpha\big(\{\sigma_i(t)\}\big) \tag{1}$$

with independent emission distribution Q_α,

$$Q_\alpha^{\text{ind}}\big(\{\sigma_i(t)\}\big) = \prod_{i}^{\text{cells}} \big(m_{i\alpha}\big)^{\sigma_i}\big(1 - m_{i\alpha}\big)^{(1-\sigma_i)}. \tag{2}$$

The model parameters are therefore the mode probabilities, w_α and the mode-dependent mean firing probability of each neuron, $m_{i\alpha}$. Models of this form may be fit by the expectation-maximization (EM) algorithm, which iteratively alternates an expectation step (E-step) with a maximization step (M-step). Each iteration of the E-step and M-step may be shown to increase the likelihood of the model.

§2.a.1. *The E-step.* In the E-step, we use the currently-estimated parameters to compute the posterior probability over the mode in each time bin, conditioned on the observed data:

$$P\big(\alpha_t \,|\, \{\sigma_i(t)\}\big) = \frac{w_{\alpha_t} Q_{\alpha_t}(\{\sigma_i(t)\})}{\sum_\beta w_\beta Q_\beta(\{\sigma_i(t)\})}. \tag{3}$$

§2.a.2. *The M-step.* We then use the estimated probability of modes in each time bin to take weighted averages over the time bins that update the model parameters. For example, the overall mode probability w_α is simply set to the average of mode α's probability in each time bin:

$$w_\alpha \leftarrow \frac{1}{(T+1)} \sum_{t=0}^{T} P\big(\alpha_t \,|\, \{\sigma_i(t)\}\big). \tag{4}$$

Likewise, the mode-dependent spiking probabilities are updated in an analogous fashion:

$$m_{i\alpha} \leftarrow \sum_{t=0}^{T} \sigma_i(t) P\big(\alpha_t \,|\, \{\sigma_i(t)\}\big) \Big/ \sum_{t=0}^{T} P\big(\alpha_t \,|\, \{\sigma_i(t)\}\big). \tag{5}$$

These two steps are then repeated for a fixed total number of iterations.

In deriving the full model, we generalized the above by adding temporal correlations in the form of probabilistic transitions between modes in adjacent time bins. This led to a more complex set of steps for computing the posterior distribution over modes in the E-step, as well as a new step for updating the transition probabilities in the M-step. We then added correlation between cells into the emission distributions $Q_\alpha(\{\sigma_i(t)\})$, which added further complexity to the M-step. Nevertheless, the overall EM form of the algorithm was preserved, as was the intuition that the M-step involves estimating parameters by taking weighted averages, weighted by the posterior distribution over modes.

As discussed in the main text, the full model better captured pairwise correlations between cells, and also captured temporal correlations (which were completely ignored by the independent mixture model). We now describe the algorithm for fitting the full hidden Markov model model with mode-dependent correlations.

§2.b. **Maximum likelihood estimation of model parameters.** We define the hidden Markov model (HMM) and introduce our notation in the main text. Briefly, we assume M distinct hidden modes, labeled by $\alpha = 1 \dots M$. For each mode, there is an emission distribution over the binary pattern $\{\sigma\}$ in a given time bin, $Q_\alpha(\{\sigma_i(t)\}) = P(\{\sigma_i(t)\} \,|\, \alpha_t)$. Finally, transitions between modes are parameterized by the matrix $T(\alpha \to \beta) = P(\beta_t \,|\, \alpha_{t-1})$ and the distribution over modes in the initial time bin is denoted $w_0(\alpha) = P(\alpha_0 = \alpha)$.

To fit the model we apply the Baum-Welch algorithm. This method alternates two steps: an expectation step (E-step) and a maximization step (M-step). These are alternated for a fixed total number of iterations.

§2.b.1. *Parameter initialization.* We set the initial transition matrix to a uniform distribution in each column, and $w_0(\alpha)$ to a uniform distribution. All tree edges were initialized to zero. The remaining parameters of the emission distributions are the mode-dependent firing probabilities for each mode and cell. It is important to choose these differently for each mode, because if two modes have identical initial parameters the fitting algorithm will never separate them. At the same time, if the initial values of any modes are too extremely different from the data, compared to other modes, they will have their probability driven to zero and effectively drop out of the model. We therefore generated the initial mean vectors randomly, but with only a small amount of variability. Specifically, each component of each mean vector was drawn randomly and independently from the uniform distribution over $[0.45, 0.55]$.

§2.b.2. *The E-step.* In the E-step, we use the currently-estimated parameters to calculate the following posterior probability distributions over mode identities at each time bin t: $P(\alpha_t \,|\, \{\sigma\}_0^T)$ and $P(\alpha_t, \beta_{t-1} \,|\, \{\sigma\}_0^T)$. Here, $\{\sigma\}_0^T$ represents the $N \times (T+1)$ matrix consisting of all of the observed spiking data, from time bins 0 through T. That is, in terms of the notation used in the main text, $\{\sigma\}_0^T \equiv \big(\{\sigma_i(0)\}, \cdots, \{\sigma_i(t)\}, \cdots, \{\sigma_i(T)\}\big)$. We may

simplify the calculation of these quantities by application of Bayes' rule, giving:

$$P_1(\alpha,t) \equiv P(\alpha_t \,|\, \{\sigma\}_0^T) = F(\alpha,t)B(\alpha,t)/Z_1(t) \tag{6}$$

$$P_2(\alpha,\beta,t) \equiv P(\alpha_t,\beta_{t-1} \,|\, \{\sigma\}_0^T) = F(\beta,t-1)B(\alpha,t)Q_\alpha(\{\sigma\}_t)T(\beta\to\alpha)/Z_2(t) \tag{7}$$

Here, we have introduced the forward and backward filtering distributions $F(\alpha,t)$ and $B(\alpha,t)$:

$$F(\alpha,t) \equiv P(\alpha_t, \{\sigma\}_0^t) \tag{8}$$

$$B(\alpha,t) \equiv P(\{\sigma\}_{t+1}^T \,|\, \alpha_t) \tag{9}$$

And $Z_1(t),\ Z_2(t)$ are simply normalizing constants. The E-step thus reduces to calculation of the forward and backward functions. Further use of Bayes' rule yields iterative equations for these quantities:

$$F(\alpha,t) = Q_\alpha(\{\sigma_i(t)\}) \sum_\beta^{\text{modes}} T(\beta\to\alpha)F(\beta,t-1) \tag{10}$$

$$B(\alpha,t) = \sum_\beta^{\text{modes}} Q_\beta(\{\sigma_i(t+1)\})T(\alpha\to\beta)B(\beta,t+1) \tag{11}$$

Together with the boundary conditions, $F(\alpha,0) = Q_\alpha(\{\sigma\}_0)w_0(\alpha)$ and $B(\alpha,T) = 1$, these equations allow F to be computed in a forward pass through all the data (Eqn. 10) and B to be computed in a backward pass (Eqn. 11). As an implementation detail, we note that naive application of these equations is numerically unstable, as they tend to drive F and B toward zero. We therefore instead compute normalized versions of F and B, applying the normalization after each time bin. These $\alpha-$independent normalizing factors are ultimately unimportant, as they may simply be absorbed into Z_1, Z_2 defined above.

§2.b.3. *The M-step.* After computing the posterior probabilities over mode identity, we may define the following function, similar to a log-likelihood, which is maximized in the M-step:

$$\begin{aligned}\mathcal{L} &\equiv \left(\sum_\alpha^{\text{modes}} \sum_{t=0}^T P_1(\alpha,t)\log Q_\alpha(\{\sigma_i(t)\})\right) + \left(\sum_{\alpha,\beta}^{\text{modes}} \sum_{t=1}^T P_2(\alpha,\beta,t)\log T(\beta\to\alpha) + \sum_\alpha^{\text{modes}} P_1(\alpha,0)\log w_0(\alpha)\right) \\ &\equiv \mathcal{L}_1 + \mathcal{L}_2\end{aligned} \tag{12}$$

The parameters $T(\beta \to \alpha)$ and $w_0(\alpha)$ only enter into $\mathcal{L}_2$, which may be analytically optimized subject to the normalization constraints $\sum_\alpha w_0(\alpha) = 1$ and $\sum_\alpha T(\beta\to\alpha) = 1$. This yields the following update rules for these parameters:

$$T(\beta\to\alpha) \leftarrow \sum_{t=1}^T P_2(\alpha,\beta,t) / \sum_\alpha^{\text{modes}} \sum_{t=1}^T P_2(\alpha,\beta,t) \tag{13}$$

$$w_0(\alpha) \leftarrow P_1(\alpha,0) \tag{14}$$

The remaining parameters define the emission distributions Q_α, and are updated by maximizing $\mathcal{L}_1$. Our model takes the form,

$$Q_\alpha^{\text{tree}}\big(\{\sigma_i(t)\}\big) = \prod_i^{\text{cells}} p_\alpha(\sigma_i) \prod_{\langle i,j\rangle}^{\text{edges}} \frac{p_\alpha(\sigma_i,\sigma_j)}{p_\alpha(\sigma_i)p_\alpha(\sigma_j)} \tag{15}$$

The parameters of Q_α are, therefore, the choice of tree topology (i.e., the set of edges $\langle i,j\rangle$), together with the pairwise distribution $P_\alpha(\sigma_i,\sigma_j)$ on each tree edge and the single-cell distribution $P_\alpha(\sigma_i)$ for each cell (these are actually somewhat redundant, since $P_\alpha(\sigma_i) = \sum_\sigma P_\alpha(\sigma_i, \sigma_j = \sigma)$ wherever i and j are connected by a tree edge). To identify the parameters, we must first choose the distributions $P_\alpha(\sigma_i)$ and $P_\alpha(\sigma_i,\sigma_j)$, and then choose the tree topology itself for each α. For the first part, $\mathcal{L}_1$ may be explicitly maximized for any fixed choice of tree edges. This yields,

$$P_\alpha(\sigma_i = \sigma) \leftarrow \sum_{t=0}^{T} P_1(\alpha,t)\delta(\sigma_i(t),\sigma)/\sum_{t=0}^{T} P_1(\alpha,t) \tag{16}$$

$$P_\alpha(\sigma_i = \sigma, \sigma_j = \sigma') \leftarrow \sum_{t=0}^{T} P_1(\alpha,t)\delta(\sigma_i(t),\sigma)\delta(\sigma_j(t),\sigma')/\sum_{t=0}^{T} P_1(\alpha,t) \tag{17}$$

Here, $\delta(\sigma,\sigma') = 1$ if $\sigma = \sigma'$, and is zero otherwise.

Finally, we must choose the tree edges. We first apply Eqn. 19 for *all* cell pairs. Then, we note that $\mathcal{L}_1$ may be expanded as follows:

$$\mathcal{L}_2 = \sum_\alpha^{\text{modes}} \left(\sum_{t=0}^{T} P_1(\alpha,t)\right)\left(\sum_{\langle i,j\rangle}^{\text{edges}} I_{ij}^\alpha - \sum_i^{\text{cells}} S_i^\alpha\right), \tag{18}$$

Where $I_{ij}^\alpha \equiv \sum_{\sigma,\sigma'} p_\alpha(\sigma_i = \sigma,\ \sigma_j = \sigma') \log \frac{p_\alpha(\sigma_i=\sigma,\sigma_j=\sigma')}{p_\alpha(\sigma_i=\sigma)p_\alpha(\sigma_j=\sigma')}$ is the mutual information between tree-adjacent cells i and j, under the distribution Q_α, and $S_i^\alpha \equiv -\sum_\sigma p_\alpha(\sigma_i = \sigma)\log p_\alpha(\sigma_i = \sigma)$ is the entropy of cell i. The only term in Eqn. 18 that depends on the tree topology is $\sum_{\langle i,j\rangle} I_{ij}^\alpha$, and so choosing the tree edges for each α is equivalent to solving the max-spanning-tree problem of finding a tree that maximizes the sum of weights along its edges. Here, the weight for edge $\langle i,j\rangle$ is I_{ij}^α. We find this tree by applying Kruskal's algorithm, which (in brief) proceeds by iteratively selecting the edge with highest weight, out of all edges that would not form a loop when added to the currently-estimated tree.

We summarize this section with pseudo-code for the fitting algorithm developed above (the complete algorithm adds to this a regularization step described below and in the main text Methods).

Initialize parameters
for $iter = 1 \ldots max\_iter$ do
▷ Begin E-step
$F(\alpha, 0) \leftarrow Q_\alpha(\{\sigma_i(0)\})w_0(\alpha)$ ▷ Begin forward pass
for $t = 1 \ldots T$ do
$F(\alpha, t) \leftarrow Q_\alpha(\{\sigma_i(t)\}) \sum_\beta T(\beta \to \alpha)F(\beta, t-1)$
$F(\alpha, t) \leftarrow F(\alpha, t)/\sum_\alpha F(\alpha, t)$
$B(\alpha, T) \leftarrow 1$ ▷ Begin backward pass
for $t = T-1 \ldots 0$ do
$B(\alpha, t) \leftarrow \sum_\beta Q_\beta(\{\sigma_i(t+1)\})T(\alpha \to \beta)B(\beta, t+1)$
$B(\alpha, t) \leftarrow B(\alpha, t)/\sum_\alpha B(\alpha, t)$
$P_1(\alpha, t) \leftarrow F(\alpha, t)B(\alpha, t)$
$P_1(\alpha, t) \leftarrow P_1(\alpha, t)/\sum_\alpha P_1(\alpha, t)$
$P_2(\alpha, \beta, t) \leftarrow F(\beta, t-1)B(\alpha, t)Q_\alpha(\{\sigma_i(t)\})T(\beta \to \alpha)$
$P_2(\alpha, \beta, t) \leftarrow P_2(\alpha, \beta, t)/\sum_{\alpha,\beta} P_2(\alpha, \beta, t)$
▷ Begin M-step
$w_0(\alpha) \leftarrow P_1(\alpha, 0)$
$T(\beta \to \alpha) \leftarrow \sum_{t=1}^T P_2(\alpha, \beta, t)/\sum_{\alpha'} \sum_{t=1}^T P_2(\alpha', \beta, t)$
$p_\alpha(\sigma_i = \sigma, \sigma_j = \sigma') \leftarrow \sum_{t=0}^T P_1(\alpha, t)\delta(\sigma_i(t), \sigma)\delta(\sigma_j(t), \sigma')/\sum_{t=0}^T P_1(\alpha, t)$
$I_{ij}^\alpha \leftarrow \sum_{\sigma,\sigma'} p_\alpha(\sigma_i = \sigma, \sigma_j = \sigma') \log \frac{p_\alpha(\sigma_i=\sigma,\sigma_j=\sigma')}{p_\alpha(\sigma_i=\sigma)p_\alpha(\sigma_j=\sigma')}$
$\{\langle i, j\rangle\}_\alpha \leftarrow \mathtt{kruskal}(\mathtt{I}^\alpha)$ ▷ Get tree edges

§2.c. **Alternative parameterization of the tree distribution.** In this section, we discuss the emission distributions; since these results hold for each mode individually we will simplify the previous notation by dropping the α sub- and superscripts on various quantities. The distribution,

$$Q_\alpha^{\text{tree}}\big(\{\sigma_i(t)\}\big) = \prod_i^{\text{cells}} p_\alpha(\sigma_i) \prod_{\langle i,j\rangle}^{\text{edges}} \frac{p_\alpha(\sigma_i, \sigma_j)}{p_\alpha(\sigma_i)p_\alpha(\sigma_j)} \tag{19}$$

may be re-parameterized as an explicit exponential family distribution:

$$Q_\alpha^{\text{tree}}\big(\{\sigma_i(t)\}\big) = \exp\left(\Gamma + \sum_i^{\text{cells}} h_i\sigma_i + \sum_{\langle i,j\rangle}^{\text{edges}} J_{ij}\sigma_i\sigma_j\right) \tag{20}$$

This latter form is more useful for certain calculations, including the regularization scheme that will be discussed below. The sum over $\langle i, j\rangle$ runs over tree neighbors; in other words, the matrix J_{ij} is nonzero only on the tree edges. The parameterization that accomplishes

this transformation is:

$$\Gamma = \sum_{i}^{\text{cells}} \log p_i(0) + \sum_{\langle i,j\rangle}^{\text{edges}} \log \frac{p_{ij}(0,0)}{p_i(0)p_j(0)} \tag{21}$$

$$h_i = (\nu_i - 1)\log\frac{p_i(0)}{p_i(1)} + \sum_{j\in\mathcal{N}(i)} \log\frac{p_{ij}(1,0)}{p_{ij}(0,0)} \tag{22}$$

$$J_{ij} = \log\frac{p_{ij}(0,0)p_{ij}(1,1)}{p_{ij}(0,1)p_{ij}(1,0)}, \tag{23}$$

where $\mathcal{N}(i)$ denotes the set of cells that are direct neighbors of i on the tree, and $\nu_i \equiv |\mathcal{N}(i)|$ is the degree of cell i.

We introduce further notation that will prove useful below: $m_i \equiv p_i(1)$ and $C_{ij} \equiv p_{ij}(1,1)$. The full distributions $p_i(\sigma_i), p_{ij}(\sigma_i,\sigma_j)$, and therefore all the parameters Γ, h_i, J_{ij}, may be expressed in terms of these quantities. In particular, we have for J_{ij}:

$$J_{ij} = \log\frac{(1 - m_i - m_j + C_{ij})C_{ij}}{(m_i - C_{ij})(m_j - C_{ij})} \tag{24}$$

From this, we can obtain a relationship that will be useful below:

$$\operatorname{sgn} J_{ij} = \operatorname{sgn} Cov(\sigma_i,\sigma_j), \tag{25}$$

where $Cov(\sigma_i,\sigma_j) = C_{ij} - m_i m_j$ and it is implicit that the equation only holds for i, j connected by a tree edge.

Finally, we note that the maximum likelihood inference of the parameters h_i, J_{ij} (Γ is a normalization constant that may be expressed in terms of the other parameters) maximizes the quantity:

$$\mathcal{L} = \Gamma(h,J) + \sum_{i}^{\text{cells}} h_i\tilde{m}_i + \sum_{\langle i,j\rangle}^{\text{edges}} J_{ij}\tilde{C}_{ij}, \tag{26}$$

where $\tilde{m}_i$ and $\tilde{C}_{ij}$ are empirical averages of σ_i and $\sigma_i\sigma_j$, respectively. Returning to the notation of the previous section, $\tilde{m}_i^\alpha = \sum_t P_1(\alpha,t)\sigma_i(t)/\sum_t P_1(\alpha,t)$, and $\tilde{C}_{ij}^\alpha$ is the analogous weighted average over $\sigma_i\sigma_j$. This turns out to be maximized when $m_i = \tilde{m}_i$ and $C_{ij} = \tilde{C}_{ij}$; h, J may then be recovered by using Eqns. 22, 23.

§2.d. L_1 **regularization.** To prevent overfitting, we imposed a regularization that penalizes the addition of tree edges. Since J_{ij} is nonzero only for tree edges, an L_1 regularization that encourages J_{ij} to be zero accomplishes this objective. Therefore, we replaced the above maximization of $\mathcal{L}$ with the maximization of $\mathcal{L} - \eta\sum_{ij}|J_{ij}|$, with $\eta \geq 0$ the regularization parameter. The optimization criterion becomes $m_i = \tilde{m}_i$ and $C_{ij} = \tilde{C}_{ij} - \eta \operatorname{sgn} J_{ij}$. Applying Eqn. 25, the latter condition may be solved to yield:

$$C_{ij} = \begin{cases} \tilde{C}_{ij} - \eta, & \widetilde{Cov}(\sigma_i,\sigma_j) > \eta \\ \tilde{C}_{ij} + \eta, & \widetilde{Cov}(\sigma_i,\sigma_j) < -\eta \\ \tilde{m}_i\tilde{m}_j & |\widetilde{Cov}(\sigma_i,\sigma_j)| \leq \eta \end{cases} \tag{27}$$

In other words, the tree edge weights are closer to zero than they would be without the regularization, and cells with too-small covariance are not connected at all. In order to incorporate the regularization step into the fitting algorithm, we must modify line 19 of the pseudo-code algorithm by first calculating m_i^α for all cells and C_{ij}^α for all cell pairs, using Eqn. 27, and then using these parameters to obtain $p_\alpha(\sigma_i, \sigma_j)$.

§2.e. **Computation of the correlation matrix in the tree model.** The tree emission distribution is explicitly parametrized by the pairwise distribution over tree-adjacent neurons. However, neurons that are not directly connected will still be correlated, due to the indirect interaction mediated by those neurons that they are mutually connected to. In this section we derive the simple formula for computing the mode-dependent correlation coefficient between arbitrary neuron pair i and j. As above, we suppress the mode index on various quantities; everything we do in this section applies within each mode independently.

Within each mode, any pair of neurons will be connected by a unique chain of tree edges (to handle the case of trees that are not fully-connected, we allow inclusion of "null" zero-weight edges in the chain). We denote the set of tree edges in this chain by $\mathcal{P}(i,j)$. Let j^* represent the neuron which is directly adjacent to j along the chain connecting i to j; i.e. $\langle j, j^* \rangle \in \mathcal{P}(i,j)$. Then neurons i and j are conditionally independent given j^*: $p(\sigma_j \,|\, \sigma_i, \sigma_{j^*}) = p(\sigma_j \,|\, \sigma_{j^*})$. We may use this to simplify the equation for the covariance between i and j:

$$\begin{aligned} Cov(\sigma_j, \sigma_i) &= \sum_{\sigma_i, \sigma_{j^*}} \mathbf{E}\left[(\sigma_j - m_j) \,|\, \sigma_j *\right] (\sigma_i - m_i) p\big(\sigma_{j^*}, \sigma_i\big) \\ &= \left(\mathbf{E}\left[(\sigma_j - m_j) \,|\, \sigma_j * = 1\right] - \mathbf{E}\left[(\sigma_j - m_j) \,|\, \sigma_j * = 0\right]\right) \sum_{\sigma_i, \sigma_{j^*}} \sigma_{j^*} (\sigma_i - m_i) p\big(\sigma_{j^*}, \sigma_i\big) \end{aligned} \tag{28}$$

The first factor, the difference of conditional expectations, turns out to equal $\frac{Cov(\sigma_j, \sigma_{j^*})}{Var(\sigma_{j^*})}$; it is simple to compute because j and j^* are directly connected. The second factor, the sum over σ_i, σ_{j^*}, is in fact $Cov(\sigma_{j^*}, \sigma_i)$. We therefore have derived a recursive formula for the covariance:

$$Cov(\sigma_j, \sigma_i) = \frac{Cov(\sigma_j, \sigma_{j^*})}{Var(\sigma_{j^*})} Cov(\sigma_{j^*}, \sigma_i) \tag{29}$$

Dividing both sides by $\sqrt{Var(\sigma_j) Var(\sigma_i)}$ we obtain a further simplification:

$$\rho(\sigma_j, \sigma_i) = \rho(\sigma_j, \sigma_{j^*}) \rho(\sigma_{j^*}, \sigma_i), \tag{30}$$

where $\rho(\sigma_i, \sigma_j)$ is the correlation coefficient: $\rho(\sigma_i, \sigma_j) \equiv \frac{Cov(\sigma_i, \sigma_j)}{\sqrt{Var(\sigma_i) Var(\sigma_j)}}$. Finally, we may iterate the above rule to obtain:

$$\rho(\sigma_j, \sigma_i) = \prod_{\langle k, l \rangle \in \mathcal{P}(i,j)} \rho(\sigma_k, \sigma_l). \tag{31}$$

That is, the correlation coefficient between any two neurons is simply the product over the correlation coefficient associated with each tree edge along the chain directly connecting

the two neurons. Interpreting the number of links in this chain as a measure of distance between the two neurons, this result implies that correlations fall off exponentially with distance. In this sense, the tree model only allows “weak” correlations.

§2.f. **Maximizing the posterior over mode sequences.** After fitting the hidden Markov model, we would like to be able to identify the most probable mode sequence $\hat{\alpha}_t = \operatorname{argmax} P(\alpha_0^T \mid \{\sigma\}_0^T)$, where the probability distribution here represents the posterior over the full mode sequence, conditioned on *all* the data. We solve this by the Viterbi algorithm, which is standard, but we review it here for completeness.

The Viterbi algorithm involves computation of the function $\mu(\alpha_t, t) \equiv \max_{\alpha_0^{t-1}} P(\alpha_0^t, \{\sigma\}_0^t)$. This is done recursively through a forward pass over the data, by applying the iterative formula:

$$\mu(\alpha, t) = Q_\alpha(\{\sigma_i(t)\}) \max_\beta T(\beta \to \alpha)\mu(\beta, t-1). \tag{32}$$

The recursion is initialized by $\mu(\alpha, 0) = \max Q_\alpha(\{\sigma_i(0)\})w_0(\alpha)$. During the same forward pass, we store a function $\hat{\alpha}(\alpha_t, t-1)$, defined by:

$$\hat{\alpha}(\alpha_t, t-1) = \operatorname*{argmax}_{\alpha_{t-1}} T(\alpha_{t-1} \to \alpha_t)\mu(\alpha_{t-1}, t-1). \tag{33}$$

This function will give the most probable value for α_{t-1}, once we know the most probable α_t. The full sequence is therefore obtained in a backward pass by the rules $\hat{\alpha}_T = \operatorname{argmax} \mu(\alpha_T, T)$, $\hat{\alpha}_t = \hat{\alpha}(\hat{\alpha}_{t+1}, t)$.

§3. Characterizing Mode Spatial Receptive Fields

For each fit Tree HMM latent mode α, the corresponding receptive field (RF) was mapped by computing the "mode-triggered average" (MTA) stimulus obtained in the presence of a white noise checkerboard stimulus. That is, by averaging all stimuli preceding a time bin in which the mode was active. For analysis of RF shape, the spatial component of the MTA was extracted using singular value decomposition (SVD) across time. Each mode spatial RF was smoothed by convolving with a two-dimensional Gaussian kernel having a standard deviation of 1 checker, and interpolated by a factor of 4. The center of each mode's spatial RF profile was identified as the spatial position corresponding with the extremal (i.e. largest absolute) RF value. To further mitigate the effects of noise, all of the following analyses were then restricted to the 29×29 interpolated checker region (i.e. 7.25×7.25 in original checker units) centered on the spatial RF center. For all modes, this window was sufficiently large enough to encompass the border, estimated visually and then verified post-hoc, of the region contiguous around the RF center outside of which RF magnitudes were below approximately 70% of the extremal value evoked in the RF center.

§3.a. **Noise & oriented dipole analysis.** For each of the 50 Tree HMM modes, we fit a $2D$ Difference of Gaussians (DoG) model via the method of least squares (using the genetic algorithm as our optimization algorithm) to the corresponding smoothed spatial RF, given by:

$$f_{\text{DoG}}(\vec{x}) = K_1 \cdot g(\vec{x}; \vec{\mu}_1, \Sigma_1) - K_2 \cdot g(\vec{x}; \vec{\mu}_2, \Sigma_2) \tag{34}$$

where $\vec{x} \in \mathbb{R}^2$, the constant scaling parameters $K_1, K_2 \in (-\infty, \infty)$, g denotes the bivariate Gaussian distribution, $\vec{\mu}_1$ and $\vec{\mu}_2$ denote the means, and Σ_1, Σ_2 denote the covariance matrices of the respective Gaussian distributions. Note that we fit an unconstrained form of the DoG model in that we allowed the means to be spatially shifted, i.e. we allowed for $\vec{\mu}_1 \neq \vec{\mu}_2$, and placed no other constraints on the parameters. For each mode spatial RF, we fit the above $2D$ DoG model a total of 100 times, using different randomly chosen initial parameter values each time. We then chose the best model fit as the one which minimized the mean squared error (MSE), and used this best model fit for subsequent analyses.

§3.a.1. *Noise analysis.* We first wanted to identify if there were any modes for which the spatial RF was predominantly noise, so that we could exclude these from subsequent analyses. By "predominantly noise", we mean that the spatial RF lacks the fundamental property of containing at least one extremum with an amplitude significantly above the baseline noise level, which we would expect a "good" spatial RF to exhibit. Note that the unconstrained $2D$ DoG model can capture both the case of a spatial RF having one extremum, and the case in which it has two extrema. If the $2D$ DoG model is a good fit in the sense that the χ^2 value equals the number of degrees of freedom, ν, and the spatial RF for each point $\vec{x}$ is independently distributed according to a normal distribution with the same noise variance - i.e. each $y_j \sim \mathcal{N}(\mu_j, \sigma_f^2)$, where y_j denotes the observed MTA value at point $\vec{x}_j$ - then:

$$\sigma_f^2 = \frac{1}{\nu}\sum_j \left(y_i - f_{\mathrm{DoG}}(\vec{x}_j;\theta)\right)^2 \equiv \mathrm{MSE} \tag{35}$$

where θ denotes the model parameters fit via least squares. That is, under these assumptions we can estimate the variance of the noise as equal to the mean squared error.

Based on this idea, we excluded a mode spatial RF if the amplitude of its greatest extremum did not exceed the estimated standard deviation of the noise for that mode, σ_f, by at least a factor of Θ (where in practice we chose $\Theta = 4.5$). That is, any mode α which had $\frac{|y_\alpha|}{\sigma_f} < \Theta$ was excluded, where y_α denotes the largest extremum of the observed spatial RF for mode α. As seen in panel B of S3 Fig, one of the 50 modes was excluded based on this criterion: mode 35. This result was consistent with visual inspections.

§3.a.2. *Oriented dipole analysis.* Next, we wanted to determine whether there were any mode spatial RFs which could be characterized as an oriented dipole. Qualitatively, a dipole RF has the fundamental characteristic of containing two extrema of opposite sign, where both extrema are significant. To quantify the presence or absence of this property, we compared the values of the two largest extrema of the best-fit DoG model. We will denote these values by V_1 (for the value of the extremum having the largest amplitude) and V_2 (for the value of the extremum having the second-largest amplitude).[1] We then classified a given mode α as having a dipole-type spatial RF if its corresponding ratio $\frac{V_2}{V_1} < \Phi < 0$, where in practice we chose the threshold value $\Phi = -0.4$. Note that in practice, for ease of visualization when compiling the global results over all 49 non-noise modes (shown in panel C of S3 Fig), we normalized each mode's spatial RF as modeled by the fit DoG model. Specifically, each spatial RF value was rescaled as:

$$\text{Rescaled } f_{\mathrm{DoG}}\left(\vec{x}_j;\theta\right) = \frac{f_{\mathrm{DoG}}\left(\vec{x}_j;\theta\right)}{\sum_k \left|f_{\mathrm{DoG}}\left(\vec{x}_k;\theta\right)\right|} \tag{36}$$

where $|\cdot|$ denotes the absolute value. Note that we chose this form of rescaling so as to preserve the relative distances between the negative and positive outliers.

As seen in panel C of S3 Fig, the above-described criterion yielded three modes (mode 16, 18 and 24) with identified dipole-type RFs. The corresponding fit DoG model (not normalized) for each of these three identified dipole-type modes is shown in Fig S3D. Note that although we did not explicitly stipulate any criterion that the two extrema should be spatially offset in our above identification procedure, we observed that for each of the three identified dipole-type mode RFs, the means of the two Gaussians of the best-fit DoG model were spatially separated (Fig S3D).

The remaining modes which did not satisfy the above ratio criterion were classified as having monopole spatial RFs. For these modes, in order for the best-fit DoG model to produce a monopole-type RF profile, either the means of the two Gaussians were nearly

[1] Note that if the two bivariate Gaussians do not significantly overlap and $K_1, K_2 \neq 0$, then the values of the two largest extrema will be $f_{\mathrm{DoG}}(\vec{\mu}_1) = K_1 \cdot g\left(\vec{\mu}_1;\vec{\mu}_1,\Sigma_1\right) - K_2 \cdot g\left(\vec{\mu}_1;\vec{\mu}_2;\Sigma_2\right)$ and $f_{\mathrm{DoG}}(\vec{\mu}_2)$.

identical as in a center-surround organization, or the second Gaussian merely contributed a negligible “noise blip”.

§3.b. **Intersection & union analysis for monopole mode RFs.** For the remaining 46 modes which had monopole spatial RFs, we next investigated how these mode spatial RFs compared to those of the individual retinal ganglion cells (RGCs). To quantify the size of mode and individual RGC spatial RFs, we first fit a single $2D$ Gaussian to each smoothed spatial RF profile via nonlinear regression (i.e. using iterative least squares estimation). As with the dipole model fits, for each mode and RGC, nonlinear regression was performed 100 times using different randomly-chosen initial parameter values, and the best-fit $2D$ Gaussian was chosen as the one with minimum MSE. An example best-fit $2D$ Gaussian is shown in S4 Fig (panel A).

We then measured the RF radius for each mode and each individual ganglion cell, which we define as the semi-major axis length of the 95% confidence interval ellipse of the best-fit $2D$ Gaussian. Under the null hypothesis of independent visual signaling, one would expect the RF of a firing pattern to approximate the union of the individual cells' RFs [1]. If we think of a Tree HMM mode as a type of definition of neural ‘codeword’ corresponding with a firing pattern, then we would likewise expect the mode RFs to approximate the weighted union of the individual RGCs which contribute to the mode. Based on this idea, we compared the actual RF radius for each mode α, which we denote by $r_\alpha^{(\text{real})}$, to the radius expected under the null hypothesis of independent visual signaling:

$$r_\alpha^{(\text{null})} \equiv \frac{\sum_{i=1}^{N} m_{i,\alpha} \cdot r_i}{\sum_{i=1}^{N} m_{i,\alpha}} \tag{37}$$

where r_i denotes the radius for cell i, and $m_{i,\alpha}$ denotes cell i's mode-dependent firing probability. Note that Eq. 37 is a generalization (to the case where the weights $m_{i,\alpha}$ are heterogeneous) of the null model formulation used in Ref. [1]. A scatter plot of $r_\alpha^{(\text{real})}$ vs. $r_\alpha^{(\text{null})}$ for each mode α is shown in panels B and C of S4 Fig.

The error bars shown in panels B and C of S4 Fig represent the standard deviation associated with each $r_\alpha^{(\text{real})}$ value, which we denote by σ_{r_α}. This value was computed via propagation of error on the fit covariance matrix parameters. Note that in practice, to ensure that the covariance matrix Σ was symmetric positive-definite, we set $\Sigma = LL^T$, where the matrix L is upper-triangular with positive values on the diagonal. We then actually adjusted the parameters L_{11}, L_{21}, and L_{22} when fitting the single $2D$ Gaussian model. Since the radius is defined to be the semi-major axis length of the 95% confidence ellipse, it follows that

$$r_\alpha^{(\text{real})} = \sqrt{5.991} \cdot \sqrt{\lambda_1} \tag{38}$$

where λ_1 denotes the largest eigenvalue of Σ. The variance $\sigma^2_{r_\alpha}$ was then estimated as the first two terms of the error propagation equation:

$$\sigma_{r_\alpha} \approx \sigma^2_{L_{11}} \left(\frac{\partial r_\alpha}{\partial L_{11}}\right)^2 + \sigma^2_{L_{21}} \left(\frac{\partial r_\alpha}{\partial L_{21}}\right)^2 \tag{39}$$

where $\sigma^2_{L_{11}}$ and $\sigma^2_{L_{21}}$ denotes the variances for L_{11} and L_{21}, respectively, estimated via our fitting procedure, and where

$$\begin{aligned}\frac{\partial r_\alpha}{\partial L_{11}} &= \frac{\sqrt{5.991}}{2} \cdot \left[\eta\left(L_{11}L_{21} - \frac{L_{11}^2}{c}\right)\right]^{-1/2} \cdot \eta\left(L_{21} - \frac{2L_{11}}{c}\right) \\ \frac{\partial r_\alpha}{\partial L_{21}} &= \frac{\sqrt{5.991}}{2} \cdot \left[\eta\left(L_{11}L_{21} - \frac{L_{11}^2}{c}\right)\right]^{-1/2} \cdot \eta L_{11}\end{aligned} \tag{40}$$

In Eq. (40), $\eta \equiv \frac{c}{acb-a^2}$, where a and b denote the first and second component, respectively, of the eigenvector of covariance matrix Σ which corresponds with the largest eigenvalue, and c and d denote the first and second component of the other eigenvector. Note that since we had interpolated each spatial RF by a factor of 4, the terms in Eq. 39 were scaled by a factor of $\frac{1}{4}$ to obtain error bar values in units of checkers (see S4 Fig, panels B and C).

§3.c. **Classification of intersection, union & independent monopole modes.** We next categorized the spatial RF of each of the 46 monopole modes as one of three mutually exclusive types: "Intersection," "Union", and "Independent". A monopole mode α was classified as having an Intersection-type spatial RF if

$$r_\alpha^{(\text{null})} - r_\alpha^{(\text{real})} > \Theta \tag{41}$$

where $\Theta > 0$ denotes a threshold criterion. Similarly, α was classified as having a Union-type spatial RF if

$$r_\alpha^{(\text{real})} - r_\alpha^{(\text{null})} > \Theta \tag{42}$$

Finally, if

$$|r_\alpha^{(\text{real})} - r_\alpha^{(\text{null})}| \le \Theta \tag{43}$$

then mode α was classified as having an Independent-type spatial RF. In practice, we tested threshold criterion values of $\Theta = \sigma_{r_\alpha}$ and $\Theta = 2\sigma_{r_\alpha}$, where σ_{r_α} denotes the standard deviation of the actual radius $r_\alpha^{(real)}$, which was calculated as previously described. To assess whether the classification of modes obtained using either of these choices for the threshold criterion was appropriate, we performed a Wilcoxon signed-rank test on the set of $\left(r_\alpha^{(\text{real})}, r_\alpha^{(\text{null})}\right)$ pairs assigned to each of the three categories.

The classification and significance results for each criterion choice are summarized in Table 1, and shown visually in panels B and C of S4 Fig. For the choice of $\Theta = 2\sigma_{r_\alpha}$: 14

modes were classified as Independent-type, which was supported by a two-sided Wilcoxon signed-rank test ($p = 0.14 > 0.05$, i.e. insignificant difference between the actual and null model radii values); 24 modes were classified as Intersection-type, which was supported by a left-tailed Wilcoxon signed-rank test ($p = 5.96 \times 10^{-8} < 0.01$, i.e. the classified modes had a significantly smaller radius than predicted by the null model); and 7 modes were classified as Union-type, also supported by a right-tailed Wilcoxon signed-rank test ($p = 0.0039 < 0.01$, i.e. the classified modes had a significantly larger radius than predicted by the null model). For the choice of $\Theta = \sigma_{r_\alpha}$: 2 modes were classified as Independent-type, which was strongly supported ($p = 1 > 0.05$); 33 modes were classified as Intersection-type, also strongly supported ($p = 1.16 \times 10^{-10} < 0.01$); and 11 modes were classified as Union-type, likewise well-supported ($p = 0.00049 < 0.01$).

Table 1. Monopole Mode RF Classification Results

	$\Theta = r_\alpha$		$\Theta = 2 \cdot r_\alpha$	
**Classification:**	**# of Modes**	**p-value**	**# of Modes**	**p-value**
**Independent**	2	1	14	0.14
**Intersection**	33	1.16×10^{-10}	24	5.96×10^{-8}
**Union**	11	0.00049	8	0.0039

§3.d. **Supplemental figures for the mode analyses.**

§3.d.1. *Supplementary Figure 3. Noise and dipole analysis results.* See pg. 17.

§3.d.2. *Supplementary Figure 4. Monopole analysis results.* See pg. 18.

References

[1] Schnitzer MJ, Meister M. Multineuronal Firing Patterns in the Signal from Eye to Brain. Neuron 2003;37:499511.

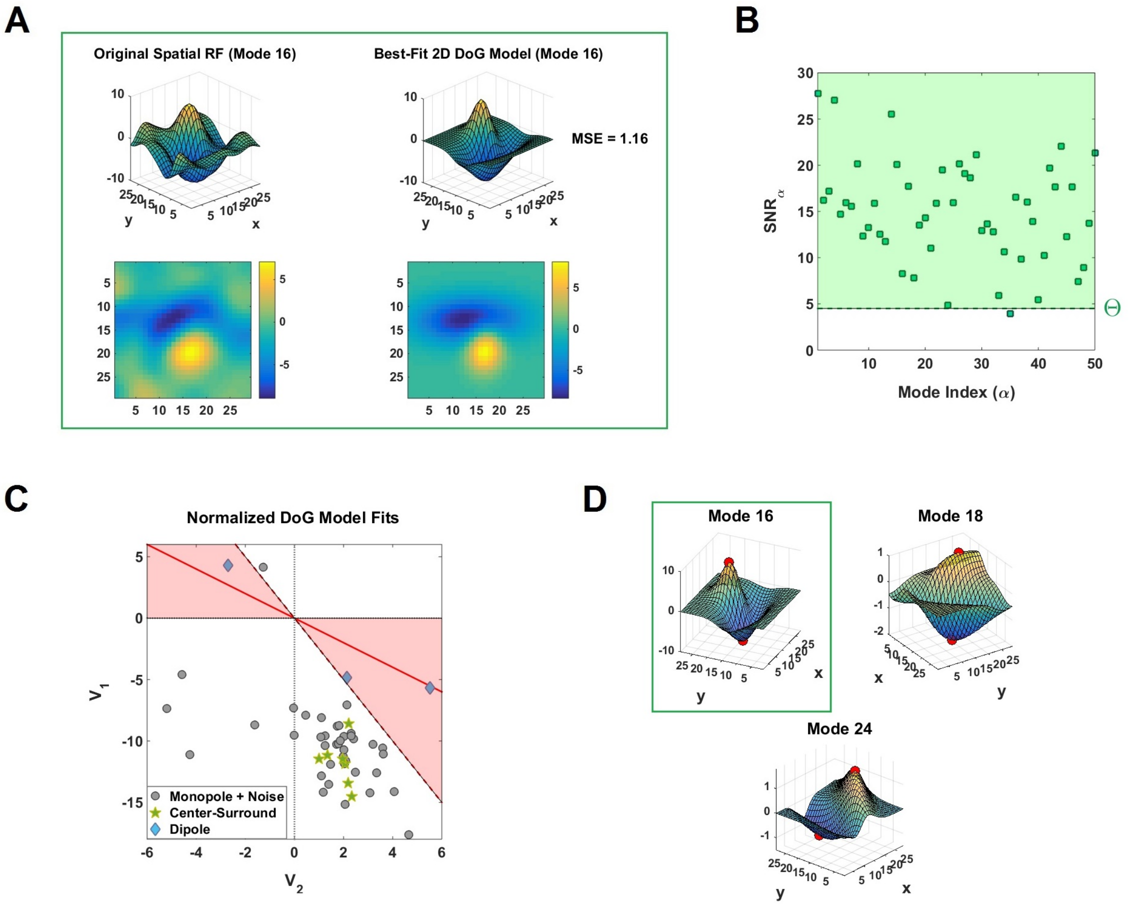

Figure S3. **Identification of noise and dipole-type mode spatial receptive fields.** (**A**) *Left*: Example smoothed spatial RF of a mode, measured by performing SVD on the mode-triggered average (MTA) stimulus. Both the $3D$ surface (top panel) and heat map representation (bottom panel) are shown. *Right*: The corresponding best-fit unconstrained $2D$ Difference of Gaussians (DoG) model for this example mode; the corresponding mean-squared error (MSE) is reported at right. (**B**) Assessment of noise modes. For each of the 50 Tree HMM modes (x-axis), we computed the ratio $\mathrm{SNR}_\alpha \equiv \frac{|y_\alpha|}{\sigma_f}$ (shown on the y-axis), where y_α denotes the largest extremum of the observed spatial RF and σ_f denotes the estimated standard deviation of the noise for mode α (see text). Green dashed line denotes the chosen threshold value $\Theta = 4.5$; ratio values above this threshold criterion are represented by the green shaded region. One mode exhibited an SNR_α value below the chosen threshold, and was consequently excluded from subsequent analyses. (**C**) Assessment of dipole-type mode spatial RFs. Each point represents one of the 49 non-noise modes. For ease of visualization, we normalized each mode's modeled spatial RF profile (see Eq. 36). We then identified the extrema of the best-fit $2D$ DoG model with the first and second-largest amplitudes via an automatic procedure. Shown is the value of the extremum with the largest amplitude (denoted "V_1", y-axis) vs. the value of the extremum with the second-largest amplitude (denoted "V_2", x-axis). Red solid line denotes the line $y = -x$, and the red dashed line denotes the bound for the chosen threshold criterion $\frac{x}{y} < -0.4$. Red shaded region denotes the region in which this criterion is met; modes within this region were categorized as having dipole-type spatial RFs (blue diamonds). For the remaining modes, the means of the two Gaussians of the best-fit $2D$ DoG model were either nearly identical as in a center-surround organization (green stars), or the second Gaussian merely contributed a negligible "noise blip" (gray circles). (**D**) Spatial RF profile, as modeled by the best-fit DoG model (not normalized), for the three identified dipole-type modes. Red dots denote the first two largest extrema for each mode.

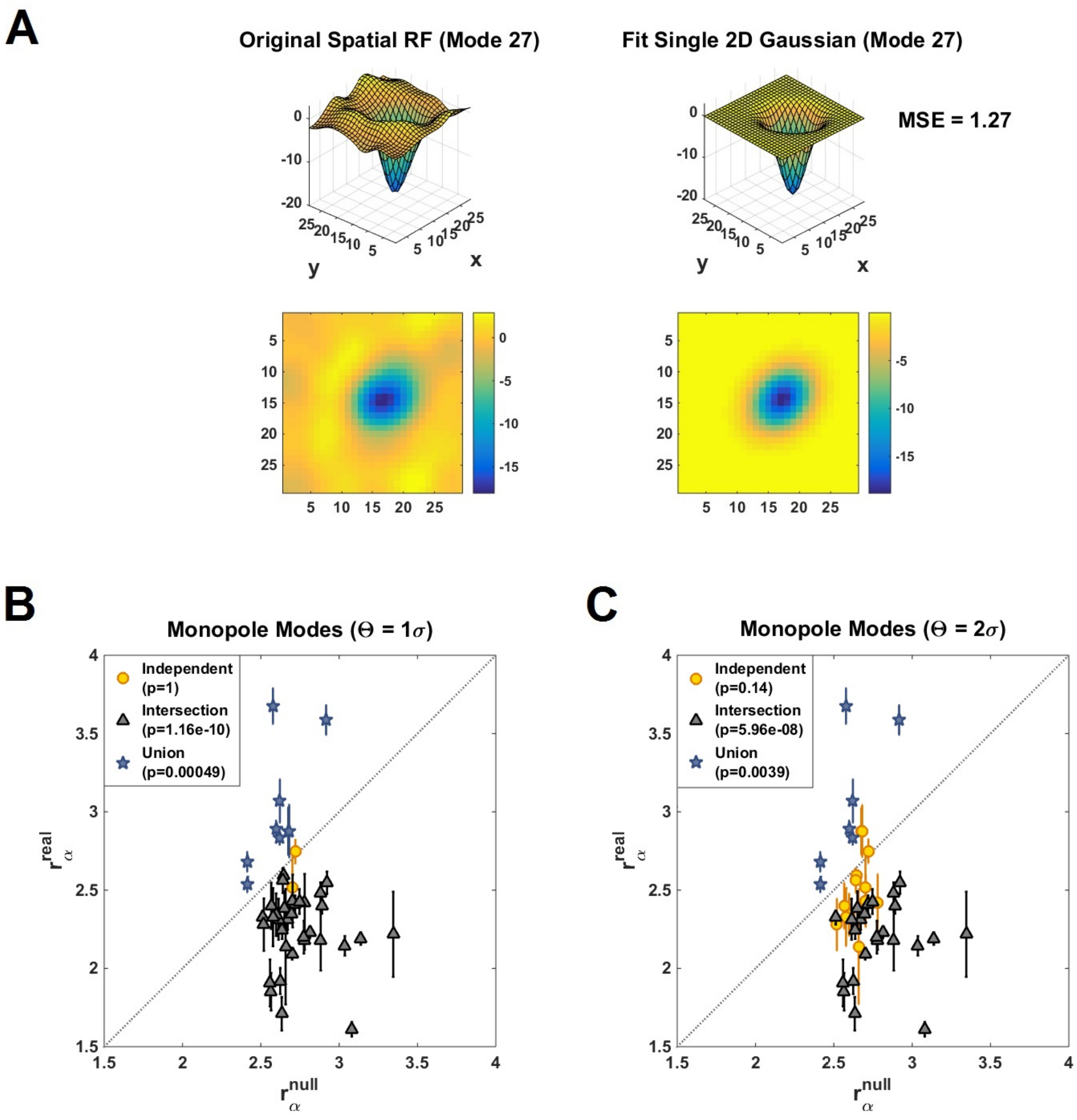

Figure S4. **Classification of monopole spatial receptive fields.** (**A**) *Left*: Smoothed spatial RF of an example monopole mode, measured by performing SVD on the mode-triggered average (MTA) stimulus. Layout as in S1 Fig. *Right*: The corresponding best-fit single $2D$ Gaussian model for this example mode; the corresponding mean-squared error (MSE) is reported at right. (**B**) Classification results obtained when the threshold criterion $\Theta = \sigma_{r_\alpha}$ was used, where σ_{r_α} denotes the estimated standard deviation of the actual radius for mode α (see Eqs. 38, 39, 40). Each point corresponds with one of the 46 modes which were previously identified as having a monopole spatial RF. Shown on the x-axis is the value of the radius predicted by the null model, $r_\alpha^{(\text{null})}$, for the given mode (see Eq. 37); the actual value of the radius, $r_\alpha^{(\text{real})}$, is shown on the y-axis. Dashed gray line denotes the line of unity. Each monopole mode was classified as either independent (yellow circles), intersection-type (black triangles), or union-type (blue stars) based on the threshold criterion. The p-value obtained upon performing a Wilcoxon signed-rank test on the set of $\left(r_\alpha^{(\text{real})}, r_\alpha^{(\text{null})}\right)$ pairs assigned to each respective category is shown in the legend (top-left). (**C**) Classification results obtained when the threshold criterion $\Theta = 2\sigma_{r_\alpha}$ was used. Layout is the same as in panel (B).
